# Adaptive potential of maritime pine under contrasting environments

**DOI:** 10.1186/s12870-023-04687-w

**Published:** 2024-01-09

**Authors:** Ricardo Alía, Jose Climent, Luis Santos-del-Blanco, Amelia Gonzalez-Arrojo, Isabel Feito, Delphine Grivet, Juan Majada

**Affiliations:** 1 Instituto de Ciencias Forestales, ICIFOR-INIA, CSIC, Madrid, 28040 Spain; 2 Forest and Wood Technology Research Centre (CETEMAS), Carbayin, 33936 Spain; 3 SERIDA, Grado, Finca “La Mata”, 33820 Spain

**Keywords:** Adaptation, Phenotypic selection, Phenotypic plasticity, Drought stress, Selection gradients, Fitness components, *Pinus pinaster*

## Abstract

**Background:**

Predicting the adaptability of forest tree populations under future climates requires a better knowledge of both the adaptive significance and evolvability of measurable key traits. Phenotypic plasticity, standing genetic variation and degree of phenotypic integration shape the actual and future population genetic structure, but empirical estimations in forest tree species are still extremely scarce. We analysed 11 *maritime pine* populations covering the distribution range of the species (119 families and 8 trees/family, ca. 1300 trees) in a common garden experiment planted at two sites with contrasting productivity. We used plant height as a surrogate of fitness and measured five traits (mean and plasticity of carbon isotope discrimination, specific leaf area, needle biomass, Phenology growth index) related to four different strategies (acquisitive economics, photosynthetic organ size, growth allocation and avoidance of water stress).

**Results:**

Estimated values of additive genetic variation would allow adaptation of the populations to future environmental conditions. Overall phenotypic integration and selection gradients were higher at the high productivity site, while phenotypic integration within populations was higher at the low productivity site. Response to selection was related mainly to photosynthetic organ size and drought-avoidance mechanisms rather than to water use efficiency. Phenotypic plasticity of water use efficiency could be maladaptive, resulting from selection for height growth.

**Conclusions:**

Contrary to the expectations in a drought tolerant species, our study suggests that variation in traits related to photosynthetic organ size and acquisitive investment of resources drive phenotypic selection across and within maritime pine populations. Both genetic variation and evolvability of key adaptive traits were considerably high, including plasticity of water use efficiency. These characteristics would enable a relatively fast micro-evolution of populations in response to the ongoing climate changes. Moreover, differentiation among populations in the studied traits would increase under the expected more productive future Atlantic conditions.

**Supplementary Information:**

The online version contains supplementary material available at 10.1186/s12870-023-04687-w.

## Background

Plant populations respond to local environmental changes following two different groups of strategies [1]. The first strategy is linked to species shifting their range which is a combination of migration (niche tracking) and demographic changes (extinction risk due to demographic decline). The second is aimed to persist in their local habitat, either by niche persistence based on phenotypic plasticity or by niche evolution based on in situ genetic adaptation. It is expected that most of the forest tree species will rely on standing genetic variation to adapt in situ as the rate of environmental change is too fast for the species to migrate [2].

Adaptive potential, i.e. the genetic variance needed to respond to selection, depends on the additive genetic variance of relevant traits, typically expressed as the heritability or evolvability of a population, and the strength of stabilizing selection [3, 4]. Other factors are related to the biological effect of the environment: phenotypic plasticity, and environmental sensitivity of selection, i.e. the change in the optimum phenotype with the environment [3], and to demographic characteristics of the species, specially census size and metapopulation structure [4].

However, traits do not vary independently. At the intra-specific level of variation, trait-covariation derived from functional, genetic or developmental disposition is also known as phenotypic integration [5]. Previous studies have shown the existence of covariation at intra and inter population levels of variation between growth, reproduction, drought tolerance, and tolerance to insect herbivory [6, 7]. Moreover, the degree of phenotypic integration may vary depending on the environment. Therefore, stressful environments may limit the possibilities of adaptive genetic change due to unfavorable correlations among key traits [8]. This is especially important in traits related to the main fitness components of the species, i.e. life cycle processes including growth, development, reproduction, and survival. Phenotypic integration of life history traits may affect evolutionary change as a result of the variation in fitness of an organism in response to a given increase or decrease in the value of a trait (i.e. a selection gradient). These selection gradients can occur both within populations [9] and among populations [10–12].

Common gardens are a useful tool to analyze the response of different genotypes to environmental changes [13–15], as they can provide performance estimates for different traits under semi-natural contrasting conditions. Replicated common gardens tests are experiments where the individuals (i.e. individual genotypes), families or populations under evaluation are planted together in different sites using a specific experimental design, thereby providing a setting to assess genetic and environmental effects for the traits under evaluation. These experiments allow the estimation of in situ adaptation to these environments [16] by using a space by time approach [1]. By combining different levels of variation in the same setting, they allow the estimation of inter and intra population levels of variation as in the case of provenance-progeny trials.

Maritime pine (*Pinus pinaster* Aiton), an ecologically and economically important species in SW Europe and NW Africa, is a good example for which common garden experiments have revealed a well-structured genetic variation for different traits, related to growth, reproduction and response to biotic and abiotic factors [17–19]. Likewise, genetic changes after artificial or natural selection have been detected within and among populations [9, 20]. These results, together with the long-term network of common gardens available for this species, provide an excellent setting to gain more insight into phenotypic plasticity, standing variation and correlations among functional traits in forest trees.

The main goal of this work was to experimentally test in a model species, maritime pine, whether tree fitness is related to differences in life-history and functional traits that affect ecological strategies [21]. We evaluated the responses of 11 populations and 119 families of maritime pine covering the western natural range of the species [22], from France to Morocco. We analyzed the standing variation, phenotypic and genetic selection gradients, in a multi-site common garden experiment established in two contrasting environments differing in productivity. We expected high values of intra-population variation, resulting in high evolvability. We also expected a trade-off between growth and other avoidance and resistance traits under more stressful conditions, and a high phenotypic integration in the low productivity site. These trade-offs would result in differences in selection gradients depending on the environment, influencing the differentiation and evolvability of the populations under future climatic conditions.

## Results

### Standing genetic variation and quantitative differentiation

**Fig. 1:**
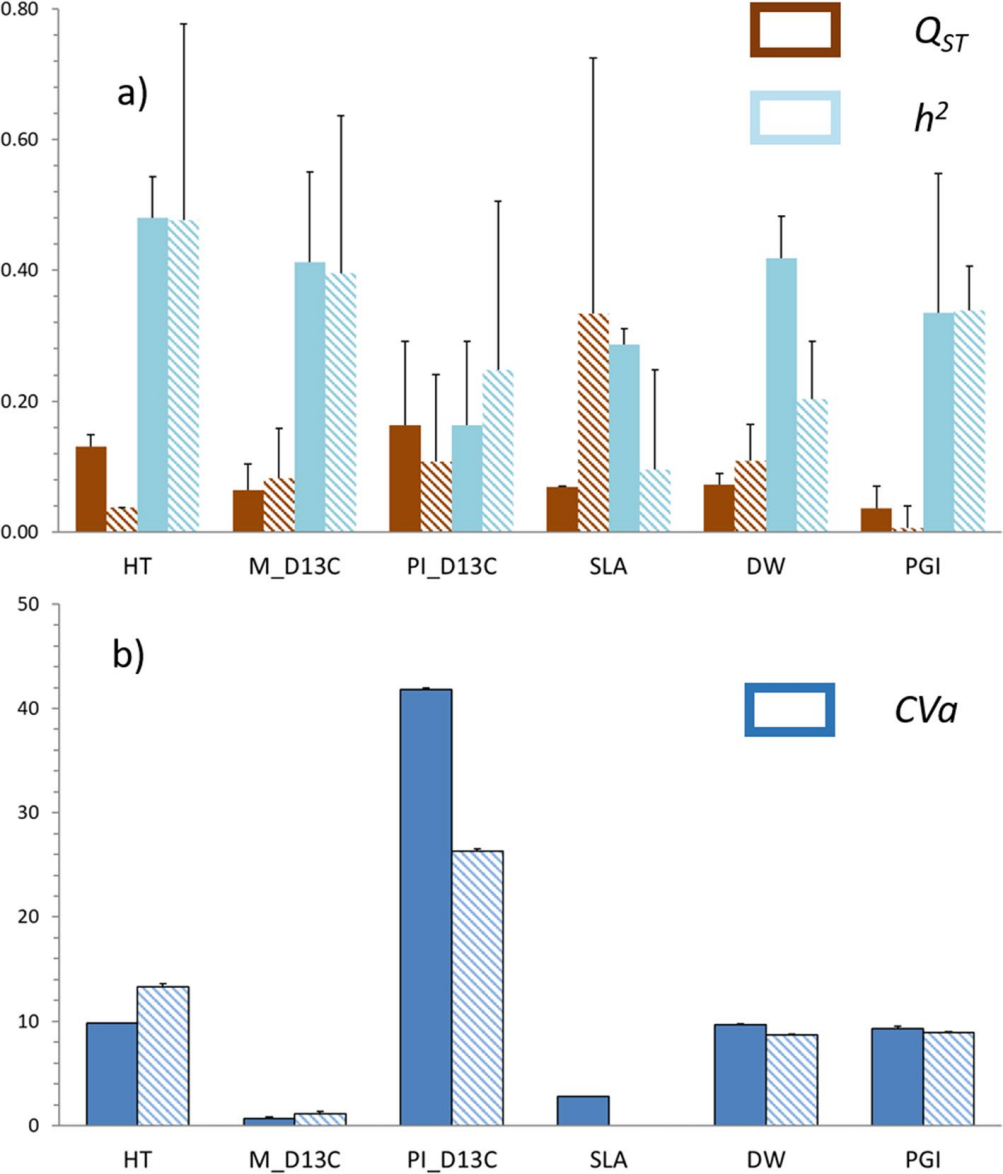
**a** Coefficient of genetic differentiation (*Q*_*ST*_), heritability (*h*^*2*^), and **b** Evolvability (CVa) of traits evaluated in maritime pine families in two common gardens (*HiProd* site –filled bars-, and *LoProd* site –hatched bars). HT: height at age 7; M_D13 C: average value of carbon isotopic discrimination; Pl_D13C: plastic response of carbon isotopic discrimination; SLA: specific leaf area; DW: leaf dry weight; PGI: phenology growth index

Most traits presented significant values of genetic variation, judged by their heritability (0.10 to 0.48) (Fig. 1, Table S1), with differences among sites depending of the trait. Height, mean δ^13^C and phenology growth index had similar heritability values at both sites. Leaf dry weight and specific leaf area had lower heritabilities at the low productivity site (*LoProd*) than at the high productivity site (*HiProd*), while the heritability for δ^13^C plasticity was higher in *LoProd* than in *HiProd*. Evolvability (CVa) differed greatly among traits, with δ^13^C plasticity showing the highest value (greater than 25%). The rest of the traits had values close to 10%, while specific leaf area and the mean δ^13^C presented values lower than 3% (Fig. 1, Table S1).

Quantitative differentiation (*Q*_*ST*_) was site- and trait-specific, and significantly lower than global genetic differentiation (*F*_*ST*_ = 0.13 with 95% confidence intervals: 0.128–0.132) for 2 out of 6 traits in the *HiProd* site (SLA and PGI) and in the *LoProd* site (HT and PGI) (Fig. 1).

### Phenotypic integration of the traits

**Fig. 2:**
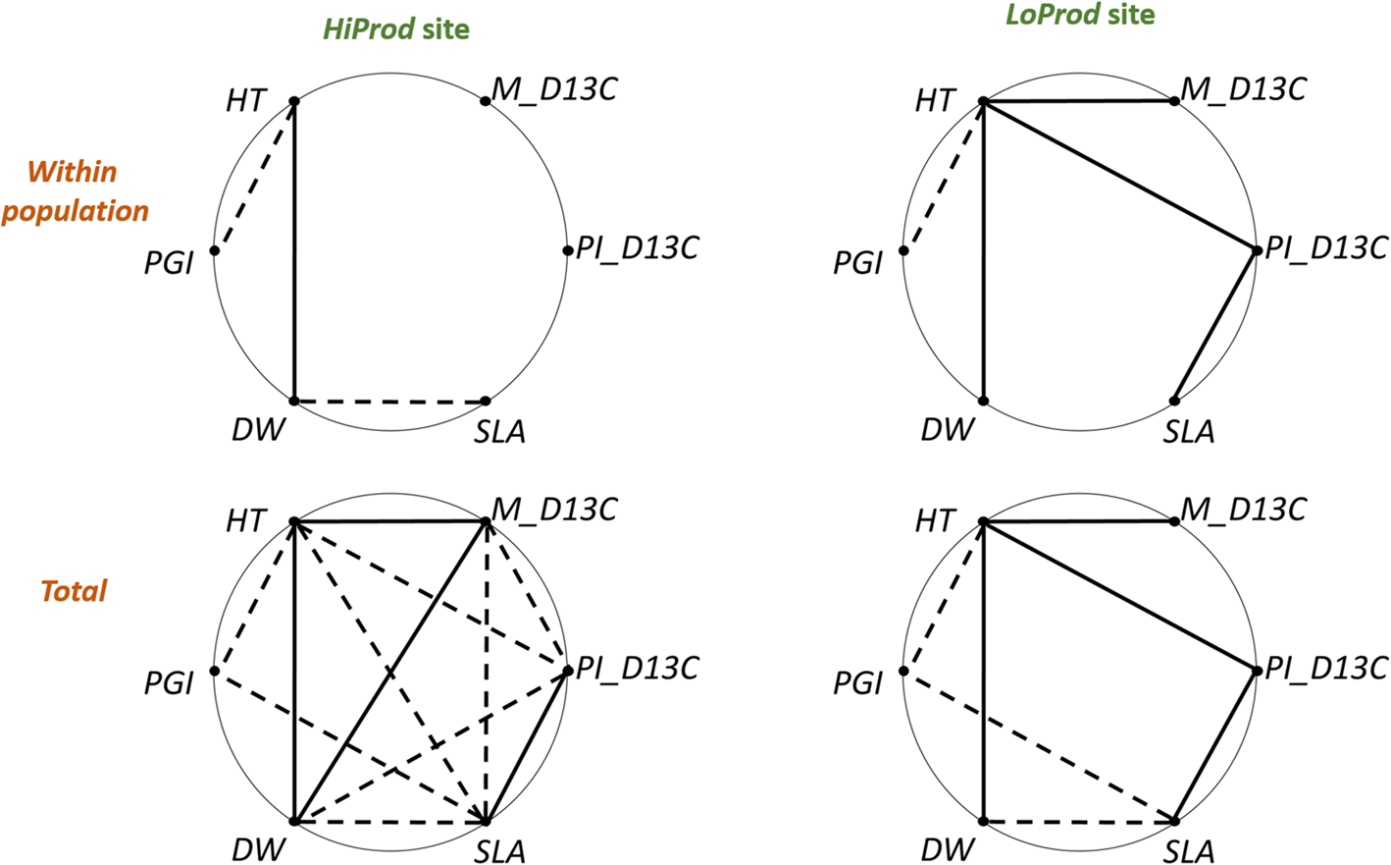
Pattern of phenotypic integration assessed in two common garden experiments differing in productivity *(HiProd* site and *LoProd* site). Within population and total correlations of estimated breeding values of the mother trees are represented. Solid lines represent significant positive correlations, and broken lines represent significant negative correlations (significance level α ≤ 0.05). [HT: height at age 7; M_D13 C: average value of carbon isotopic discrimination; Pl_D13C: plastic response of carbon isotopic discrimination; SLA: specific leaf area; DW: leaf dry weight; PGI: phenology growth index]

Phenotypic integration at the within population level of variation was higher at the *LoProd* site, but the difference to the *HiPro*d site was not statistically significant (Table 1). However, when considering the total breeding values, phenotypic integration was significantly higher in the *HiProd* site. HT was significantly correlated (both within population and total) with leaf dry weight (positive) and with phenology growth index (negative) at both sites (Fig. 2). Moreover, there were notable differences between the two sites in the correlations between HT and δ^13^C mean and plasticity. These correlations were positive at the *LoProd* site, but were not significant at the within population level of variation and negative with δ^13^C plasticity (as SLA and RGH) at the *HiProd* site.

**Table 1:** Corrected and relative degree of phenotypic integration (*PINT*) among six traits in two common gardens in maritime pine

Site^a^	Level of variation	Corrected *PINT* ± SE (99% CI)^b^	Relative *PINT*
***HiProd***	Within population	0.175 ± 0.003 ns (0.127,0.317)	4.347 ns
	Total	0.875 ± 0.013 *** (0.554, 1.273)	18.343***
***LoProd***	Within population	0.346 ± 0.017 ns (0.152, 0.777)	8.969 ns
	Total	0.342 ± 0.017 ns (0.185, 0.791)	8.881 ns

*ns* non significant

^a^*HiProd *High productivity site (site index 22), *LoProd *Low productivity site (site index 6)

^b^SE Standard error, *CI *Confidence interval

*** α ≤ 0.001

### Selection gradients

Significant and positive genetic selection gradients (*β*_G_) were found for dry leaf weight at both sites, while a significant and negative genetic selection gradient for phenology growth index was observed at the *HiProd* site. At the within population level, linear phenotypic selection gradients were significant for most traits, except for δ^13^C plasticity, specific leaf area and phenology growth index at the *LoProd* site (Table 2). Significant quadratic phenotypic selection gradients were found for dry leaf weight and phenology growth index at both sites.

The inclusion of population effects in the estimates slightly modified the outcomes, with non-significant linear selection gradients for δ^13^C plasticity at any site, and for mean δ^13^C at the *LoProd* site, while *SLA* and phenology growth index showed significant linear selection gradients at *LoProd* site (Table 2). For PGI, mean δ^13^C and *SLA*, the quadratic term of the selection gradients was positive at *HiProd* site. It is interesting to notice that the sign of the linear selection gradient was the same as for the two levels of variation analyzed (within population and total), as well as for the genetic selection gradients.

**Table 2:** Genetic selection gradients (*β*_*G*_), linear and quadratic phenotypic selection gradients within populations (*β*, *γ* respectively), linear and quadratic total phenotypic selection gradients (*β*_P_,*γ*_P_ respectively), respect to height as a fitness *proxy* at the two sites (in bold, significance level α ≤ 0.05)

		Geneticc selection gradient	Phenotypic selection gradient			
		Intra population	Intra population		Total	
Trait	Site	*β*_G_	*β*	*γ*	*β*_P_	*γ*_P_
**M_D13C**	*HiProd*	-0.041 (*0.046*)	**-0.030 (0.011)**	ns^a^	**-0.045 (0.015)**	**-0.032 (0.012)**
	*LoProd*	0.059 (*0.049*)	**-0.042 (0.016)**	ns	ns	ns
**PI_D13C**	*HiProd*	0.104 (*0.134*)	**0.027 (0.010)**	ns	ns	ns
	*LoProd*	0.197 (*0.136*)	ns	ns	ns	ns
**SLA**	*HiProd*	-0.042 (*0.070*)	**0.132 (0.012)**	ns	**0.135 (0.012)**	**-0.026 (0.008)**
	*LoProd*	ns	ns	ns	**0.087 (0.022)**	ns
**DW**	*HiProd*	**0.182** (*0.042*)	**0.232 (0.011)**	**-0.032 (0.007)**	**0.389 (0.021)**	**-0.062 (0.013)**
	*LoProd*	**0.213** (*0.059*)	**0.383 (0.021)**	**-0.035 (0.011)**	**0.496 (0.029)**	**-0.056 (0.011)**
**PGI**	*HiProd*	**-0.145** (*0.071*)	**-0.046 (0.010)**	**-0.026 (0.007)**	**-0.051 (0.012)**	**0.011 (0.006)**
	*LoProd*	-0.173 (*0.216*)	ns	**-0.030 (0.011)**	**-0.025 (0.015)**	ns

^a^*ns* Parameter non-significant according to the log-linear model

## Discussion

By assessing the response of range-wide populations of maritime pine in two contrasted sites, we detected high values of additive variance, different selection gradients for functional traits and degrees of phenotypic integration, characteristics needed to forecast the adaptive potential of populations under climate change [23].

Our two common garden sites are located within the natural distribution of the species in the Atlantic region and the populations span the whole geographic range of the species (See Methods S1). Despite their geographical proximity and similar mean temperature and rainfall, both environments differed greatly in productivity, displaying values at both the extremes of the entire range of the species in Spain [24]. These differences in productivity could be mainly related to soil water availability, according to a negative relationship between site index and δ^13^C in the same species and in the same region [25].

In this study, we used height as a surrogate of fitness (Methods S2). At the age of the experiments (7 years) we were also able to measure other fitness-related traits (diameter in the two experiments, and male and female flowering in the high productivity site -the only one reaching a reproductive stage). These fitness related traits displayed high values of variation across populations as previously shown [19]. The results obtained using diameter and flowering are concordant with those presented for height in the present study as well as in other trees species, reinforcing the use of height as a fitness surrogate.

Our study focuses on two different levels of genetic variation: within-population and total. These two levels of variation are expected to affect differently the phenotypic integration and the variation among sites [26], the plastic responses [27], and the adaptability of individuals and families to different environments. Next, we discuss the outcomes of our study at the two considered levels of variation.

### Within-population patterns

We report high values of standing genetic variation and phenotypic variation within populations for the six traits analyzed (see Fig. 1), which would allow mid-term adaptation to future climatic conditions through genetic change [2, 16], as predicted from the breeder´s equation [28]. Moreover, within-population variation was very high compared to differentiation among populations, following a general pattern in forest trees [16, 29].

The degree of phenotypic integration should be determined by the genetic architecture of fitness-related traits that in maritime pine follows the polygenic adaptation model [30]. However, it is still unclear whether pleiotropy plays a major role in determining the multi-trait association as in other species [31].

Consistent with the life history theory, we found that height traded-off with avoidance/tolerance traits (isotopic discrimination, shoot growth phenology, needle biomass) under more stressful conditions. The mean and plasticity of carbon isotopic discrimination have also an adaptive value in the low productivity site. The sign of these correlations indicates an adaptive value of early growth phenology and higher needle biomass (see [32]) to avoid summer drought period and the production of more tolerant needles to physical hazards or herbivory [33, 34].

According to expectations, we found that phenotypic integration in general was higher at the low productivity site (Fig. 2). Noteworthy is the significance of within population selection gradients obtained by regression for most traits, probably as a result of the precision of the methods applied [35]. We found negative quadratic selection gradients both in dry leaf weight and phenology growth index in the two sites. The value of the selection gradients for mean δ^13^C was low at the two sites, despite the high heritability of the trait and the higher differentiation in the low production site(as in [17]). Despite the widely documented importance of drought stress as a selection driver in pines [36–38], mainly under more stressful conditions [17, 39], our results suggest that within population selection in maritime pine is not driven by water use efficiency but to a major investment of resources (needles biomass, SLA) and avoidance mechanisms of cold tolerance (e.g. late flushing). This interpretation is consistent with a low signal of selection for cavitation related traits [40] and the overrepresentation of SNP-climate correlations with winter temperatures [22]. From an ecological perspective, however, differences in precipitation determine large part of the variation within the range of the species (see Methods S1).

### Total phenotypic patterns

As expected, these intra-population trade-offs will influence the differentiation and evolvability of the populations under future climatic conditions. To understand these interactions, we need to consider population-specific evolutionary characteristics, and the relationships among traits [12, 41]. Differentiation between populations was lower than the neutral expectation, but not statistically different for most of the traits at both sites. The pattern of three traits (PGI at both sites, HT at the low productive site, and DW at the high productive site) can be interpreted as the result of stabilizing selection, that have been reported in xylem susceptibility to cavitation [42]. Lower genetic differentiation for height in low productivity sites is in accordance with other results in maritime pine [30, 43].

According to the expectations based on a generalized response syndrome to ‘stressful’ environments [44], phenotypic integration within population was higher in the low productivity site, but this pattern is not observed when considering the total phenotypic pattern (Table 1). Phenotypic integration was higher at the more productive site, where δ^13^C plasticity and specific leaf area were negatively correlated with total height, and could be regarded as maladaptive (according to the interpretation in [45]) derived from the costs that vary in magnitude depending on environmental conditions [46].

Most of the examined traits showed significant selection gradients except plasticity and mean isotopic discrimination at the low production site (Table 2), in agreement with results in the species [22, 47, 48]. The consistency of the sign of the linear selection gradient at the two levels of variation analyzed (within population and total), and those of the genetic selection gradients suggest a similar adaptive pattern at different scales in the species.

### Implications for the adaptability of the species under future climates

It has been shown that the evolutionary responses of perennial species can be constrained in unsuitable areas because adults produce maladapted offspring [49]. According to some expectations, the Atlantic area under study will increase its productivity [50] and future suitability, while in the southern range of the species the suitability will decrease [51]. Moreover, the predicted changes in the distribution of the species under future climatic conditions, indicate that the Atlantic area could be part of the ecological niche of southern genetic groups of the species [52, 53]. Under these Atlantic environmental conditions it can be expected a higher degree of phenotypic integration and differentiation between populations. These results have implications in the movement of seeds and plants such in assisted migration or climate-adjusted seed sourcing [54, 55] of southern material to favor natural selection at the within and among population levels. The lower importance of selection at the among-population level, would reduce the value of seed sourcing strategies based on the inclusion of different gene-pools to favor adaptation. Also for a large part of the actual range of the species, it would be difficult to find any southern or more xeric population from which to get migrants.

Globally, species niche models integrating infra-specific information suggest a lower reduction in the expected distribution of the species [52], advocating for the integration of information at the population/local/regional levels. It is necessary to consider the adaptive potential of the populations, which in maritime pine does not represent a limiting factor. These considerations are essential for the conservation and sustainable use of a species genetic resources that aimed to favor adaptation [56]. In this context, the importance of phenotypic plasticity and the differences among populations should be taken into consideration to forecast the effects of climate change on the future distribution of the species [14, 53, 57].

## Conclusions

Contrary to the expectations in a drought tolerant species, our study suggests that variation in photosynthetic organ size and acquisitive economics drive phenotypic selection across and within maritime pine populations. The contrasting pattern detected for total and within-population phenotypic integration, allows to forecast future trends. In particular, between-population differentiation would increase under the more productive Atlantic conditions.

## Methods

### Experimental design

We used eleven populations sampled across the major gene pools of the species [22] and covering the western range of the species (Methods S1): French Atlantic (2 populations), Iberian Atlantic (3 populations), Central Spain (4 populations), Southern Spain (1 population) and Morocco (1 population). At each population, we collected seeds from individual mother trees which were distant more than 50 m from each other, resulting in half-sib families [58].

**Fig. 3:**
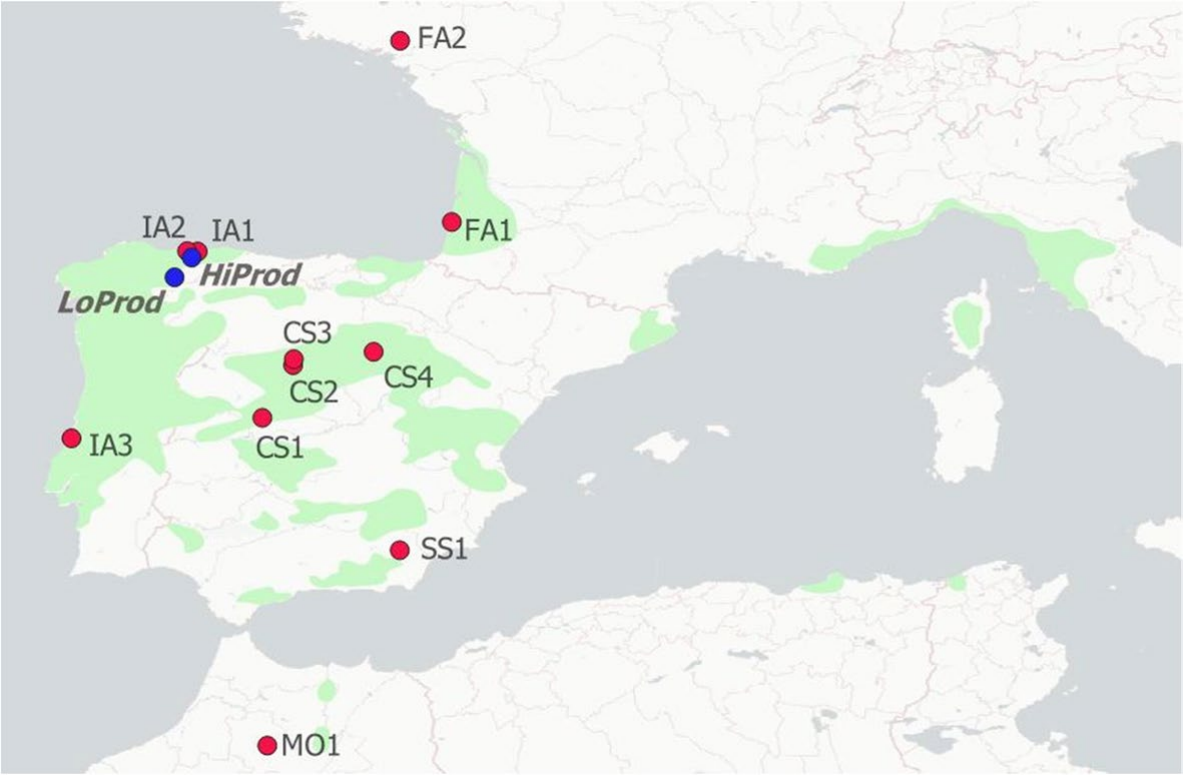
Location of the populations and the two common gardens (*HiProd* and *LoProd* sites) of maritime pine (Distribution map: CC by 4.0, www.euforgen.org)

The seedlots (identified by the mother tree) were tested at two common-garden with contrasting productivity (Fig. 3, Methods S1, Table S1.1). The High productivity *s*ite (*HiProd*) had a three and a half fold productivity than the low productivity site (*LoProd*) estimated by their site index (i.e. mean tree height in the site at age 20 [59]). The common gardens were established by late 2004, with nursery-produced one-year-old seedlings. The field design followed a row-column design with four replicates (blocks), and 4-seedling plots. We sampled up to 8 trees per family for a total of 119 families and 947 trees in the *HiProd* site, and 49 families and 375 trees in the *LoProd* site (Table S2). Forty-four of these families were present at both sites.

### Measured traits

We measured height at age 7 (HT, cm) as a fitness proxy. This trait is linked to competitive vigour [60], tree fecundity [19], and shows trade-offs at the interspecific level with tolerance or avoidance environmental stress [61] (Supplementary Methods S3 for more details).

We also recorded a set of 5 phenotypic traits informative about ecological strategies of plants [21, 62]. We measured carbon isotopic discrimination −δ^13^C− indicative of water use efficiency (see Supplementary Methods S2 for details), in the 5th and 6th growing season. We considered two different traits [63]: (i) the average value (M_D13C), and (ii) the plastic response (PI_D13C) between the two years. The plastic response was computed as the value of the year with higher water availability (5th growing season) minus the value of the year with lower water availability (6th growing season) [64].

We measured leaf traits related to trade-offs between conservative vs. acquisitive investment of resources and size [65]: (iii) Leaf dry weight (DW, mg) reflect photosynthetic organ size. It was averaged across 10 needles and two sampling years. (iv) Specific leaf area (SLA −mm^2^mg^−1^) is indicative of acquisitive investment of resources. It was estimated as the ratio between needle area and dry weight, averaged across 10 needles sampled on the 6th growing season. Needles were collected for isotopic discrimination analysis, SLA and DW estimation in mid-August in two consecutive years (during the 5th and 6th growing season of the trees).

 (v) Phenology growth index (PGI) is indicative of avoidance mechanism to summer drought as it is related to the Julian day the tree reaches the maximum daily shoot growth rate (see Methods S2). This trait was estimated as the ratio of growth at date 15/04 to the total annual height growth during the 4th growing season [30], a year with average precipitation and temperature (see Methods S1).

### Variation of traits between populations and families

We estimated population and quantitative genetic differences in HT and the other five variables (M_D13C, PI_D13C, DW, SLA, PGI) at the two sites. Given the field design the following mixed model was declared:

$$\varvec{y}={\varvec{X}}_{1}\varvec{b}+{\varvec{Z}}_{1}\varvec{p}+{\varvec{Z}}_{2}\varvec{f}+{\varvec{Z}}_{3}\varvec{r}+{\varvec{Z}}_{4}\varvec{c}+ \varvec{\varepsilon }$$

where *y* is the vector of observations for a given trait, *b* is the vector of fixed block effects, *p* is the vector of random population effects, *f* is the vector of random genetic effects of mother trees within population, *r* is the vector of rows, *c* is the vector of columns within blocks and *ε* is the vector of residuals. We estimated a variance for each random effect, where σ^2^_*p*_ is the genetic variance between populations, σ^2^_*f*_ is the genetic variance between mother trees nested within a population, σ^2^_*r*_ and σ^2^_*c*_ are the rows and column variance in the field design, and σ^2^_*e*_ is the residual variance. Bivariate models were also used to estimate covariance components among each pair of traits.

Variance and covariance components were estimated by restricted maximum likelihood (REML) using the ASREML software [66]. Population was considered as a random effect to draw inferences at the species level and to obtain an unbiased estimate of additive genetic variance, heritability and genetic population differentiation [67]. Within population additive variation was calculated (${\varvec{\sigma }}_{\varvec{a}}^{2}=4{\varvec{\sigma }}_{\varvec{f}}^{2}$) assuming half sib families [58].

### Genetic parameters

Heritability (*h*^2^) was estimated from the within population family variation assuming half-sib families, and evolvability (*CV*_*a*_)− i.e. the coefficient of additive genetic variation [68] - for each trait and site.

$${\varvec{h}}^{2}= \frac{{\varvec{\sigma }}_{\varvec{a}}^{2}}{{\varvec{\sigma }}_{\varvec{f}}^{2}+{\varvec{\sigma }}_{\varvec{e}}^{2}};{\varvec{C}\varvec{V}}_{\varvec{a}}= \frac{{\varvec{\sigma }}_{\varvec{a}}^{2}}{\stackrel{-}{\varvec{X}}}$$

Population effects were not included in the heritability calculation. Genetic differentiation between populations for quantitative traits (Q_*ST*_) was calculated from the ratios between the variances within and between populations [69–71].

$${\mathbf{Q}}_{\varvec{S}\varvec{T}}=\frac{{\varvec{\sigma }}_{\varvec{p}}^{2}}{{\varvec{\sigma }}_{\varvec{p}}^{2}+8{\varvec{\sigma }}_{\varvec{f}}^{2}}$$

The standard deviation of quantitative genetic differentiation coefficient, and heritability were calculated by the delta method [28]. A global *F*_*ST*_ estimate for the eleven populations was computed by bootstrapping across loci (1000 bootstrap iterations) based on single nucleotide polymorphism (SNPs) available from a previous study (see [30] for details).

### Within population and total breeding values for the traits

Breeding values of the mother trees for height and the 5 phenotypic traits were obtained as the BLUPs (Best linear unbiased predictors of individuals and populations- [28]) from the mixed model described above (see 5.3). We obtained two estimates: firstly, a within population breeding value (i.e. value excluding the population effect as natural selection appears to occur within each population), secondly, the total breeding value (i.e. value including the population effect, in order to take selective effects between populations into account).

### Variation in the magnitude and patterns of phenotypic integration

We estimated the correlation matrices among traits for the two estimated breeding values (within population and total, see 5.5), and for each of the two sites. From the correlation matrices the index of phenotypic integration (corrected *PINT*) was computed as the relative variance of eigenvalues [72, 73]. This value is corrected by subtracting the expected amount of integration produced by random covariation, determined by (N − 1) / n, where N is the number of traits, and n the number of individuals. A higher *PINT* indicates that variation is more concentrated among some traits, which means integration is higher. The relative index of phenotypic integration (Relative *PINT*) was computed as the magnitude of the phenotypic integration expressed as a percentage of the maximum possible integration value. The statistical significance of the integration indices among the two sites was assessed by randomization. Analysis were conducted in R using the PHENIX package [74].

### Selection analysis and adaptive value of the traits

Genetic selection gradients (*β*_G_) were computed as the genetic covariance between fitness and each of the five traits, divided by the genetic variance of the trait. This method based on the bivariate mixed model described above (see 5.3), increases the precision of the estimates [35].

We also estimated linear (*β, β*_P_) and quadratic (*γ, γ*_P_) selection gradients for the two sets of breeding values (within population and total, respectively, see 5.5) and for each of the two sites. The selection gradients are estimate as the vector of partial regression coefficients of relative individual fitness on the five traits (M_D13C, PI_D13C, DW, SLA, PGI). The following log-linear model was used [75]:

$$\varvec{L}\varvec{n} \left({\varvec{\lambda }}_{\varvec{k}}\right)=\sum _{1}^{5}({\varvec{\beta }}_{\varvec{i}}{\mathcal{Z}}_{\varvec{i}\varvec{k}}+{\varvec{\gamma }}_{\varvec{i}}{\mathcal{Z}}_{\varvec{i}\varvec{k}}^{2})$$

Where λ_k_ refers to the relative individual height, i.e. the absolute fitness divided by the mean fitness in each site. The subscripts (1 to 5) refer to each of the five traits. The coefficients *β*_i_ are analogous to linear selection gradients, and the *γ*_i_ are analogous to quadratic selection gradients as defined by Lande and Arnold (1983). Statistical significance of the selection gradients was estimated using likelihood-ratio tests, by subtracting the log-likelihood for the model excluding each parameter, one at a time, from the log-likelihood of the full model, which is asymptotically chi-square distributed with one degree of freedom. Breeding values of the traits were standardized to facilitate interpretation of results [76].

Residuals of the models by means of the DHPlottinARMa R package are presented in Supplementary Methods S4.

### Supplementary Information

**Additional file 1.**

**Additional file 2.**

**Additional file 3.**

**Additional file 4.**

**Additional file 5.**

**Additional file 6.**

**Additional file 7.**

## Data Availability

The datasets analyzed during the current study are available in the Zenodo repository (DOI: 10.5281/zenodo.10260336).

## Abbreviations

CVa: Evolvability
DW: Leaf dry weight
F_ST_: Coefficient of differentiation
h^2^: Heritability
HiProd: High productivity site
HT: Height at age 7
LoProd: Low productivity site
M_D13C: Average value of isotopic discrimination in two consecutive years
PGI: Phenology growth index
PI_D13C: Plasticity index of isotopic discrimination between the two years
PINT: Coefficient of phenotypic integration
Q_ST_: Coefficient of quantitative differentiation
SLA: Specific leaf area
*β*: Linear phenotypic selection gradients within populations
*β*_*G*_: Genetic selection gradients
*β*_P_: Linear total phenotypic selection gradients (including population effect)
*γ*: Quadratic phenotypic selection gradients within populations
*γ*_P_: Quadratic total phenotypic selection gradients (including population effect)
δ^13^C: Carbon isotopic discrimination
