# Supplementary material for: Adaptive potential of maritime pine under contrasting environments
Source: BMC Plant Biol. 2024 Jan 9;24:37. doi: 10.1186/s12870-023-04687-w (PMC10775667; doi:10.1186/s12870-023-04687-w)
Supplement: Supplementary file 1 — Additional file 1. [file 12870_2023_4687_MOESM1_ESM.pdf]

Methods S1. Fitness related traits in *Pinus pinaster* Ait. common garden experiments

We measured two common gardens, with contrasting productivity, measured by a parameter related to plant size: *Hiprod* site (site index of 22 m) and *LoProd* (site index of 6 m). These contrasting differences are also found in the response of *Pinus pinaster*. The mean values in δ13C followed the climatic differences among sites (0.80 units lower in the *HiProd* site) and years (6th growing season showed lower values in δ13C (Methods S2). Also, the values in height (3 times higher in the *HiProd* site); leaf dry weight (2 times higher in the *HiProd* site), and δ13C plasticity (half of the value in the HiProd site) follows the same pattern. The other two traits (SLA and SGP) were less affected by site differences (Table S1). Plant size has been documented to be related to both age of first reproduction and total cone production, in terms of mass and number in pines, at both interspecific (Guyette et al. 2004) and intraspecific levels (Greenberg 2000). Height has also been considered as a fitness surrogate in other trees species as *Quercus* (e.g. Ramírez-Valiente et al.(2019)

In our study we evaluated other fitness related traits. Diameter at breast height (**DBH**, mm, indicative of tree fecundity) in the two sites, and two reproductive traits (Santos-del-Blanco et al. 2012) at the *HiProd* site: female reproduction (**FR**- obtained as the number of cones per tree counted at age 7) and male reproduction (**MR** -estimated by counting strobili production. At the *LoProd* site reproduction was almost totally absent (Table S1.1). Height and Diameter have lower genetic differentiation and higher heritability than the reproduction traits in the *HiProd* site.

Table S1.1. Mean, coefficient of genetic differentiation (Q_{ST}) heritability (h^2) and evolvability (CV_A) of fitness related traits in a Maritime pine genetic test evaluated in two sites. In brackets, standard error of estimates.

	HiProd				***LoProd***			
	**Mean**	Q_{ST}	h^2	CV_A	**Mean**	Q_{ST}	h^2	CV_A
**HT**	318.9	0.122 (0.061)	0.749 (0.154)	9.8	110.3	0.037 (0.043)	0.599 (0.244)	13.3
**DBH**	43.58	0.069 (0.040)	0.669 (0.141)	16.2	21.91	0.005 (0.030)	0.436 (0.228)	0.1
**MR**	31.16	0.414 (0.210)	0.161 (0.112)	19.4	-	-	-	-
**FR**	2.442	0.285 (0.177)	0.143 (0.097)	23.3	-	-	-	-

The values of the genetic correlations were always positive and significant among all the traits (Table S1.2).

Table S1.2. Genetic correlation (r_G) among fitness related traits in two sites. *HiProd* site (below diagonal), and *LoProd* site (above diagonal).

	HT	DBH	MR
**HT**	-	0.9529 (0.0457)	-
**DBH**	0.9809 (0.0174)	-	-
**MR**	0.6721 (0.2123)	0.7572 (0.2383)	-
**FR**	0.4090 (0.1657)	0.5457 (0.1294)	0.5490 (0.2352)

The values of the different parameters indicating selection (Table S1.3) are similar when considering height or the other fitness traits in the *HiProd* site (the only where reproduction could be measured).

Table S1.3. Genetic correlation (r_G), genetic selection gradients (β) and Correlation among BLUPs estimates of the families (r_{POP}) –including the population effect– of the traits respect to fitness related traits in the HiPro site (**in bold**, significant α=0.05).

**Trait**		**HT**	**FR**	**MR**
**M_D13C**	r_G	-0.1520 (*0.1681*)	-0.1550(*0.2116*)	-
	β	-0.0415(*0.0464*)	-0.0975(*0.1342*)	0.0292(*0.1054*)
	r_{POP}	**0.4398** (*0.1152*)	**0.7160**(*0.0696*)	**0.5289**(*0.1028*)
**PI_D13C**	r_G	0.2100(*0.2567*)	0.2380(*0.3401*)	-
	β	0.1037(*0.1339*)	0.2649(*0.3989*)	0.1028(*0.2459*)
	r_{POP}	**-0.4394**(*0.1152*)	**-0.6986**(*0.0731*)	**-0.5354**(*0.1019*)
**SLA**	r_G	-0.1140(*0.1866*)	0.2240(*0.2375*)	-
	β	-0.0425(*0.0702*)	-0.1988(*0.2159*)	0.2125(*0.1672*)
	r_{POP}	-0.2237(*0.1357*)	-0.3763(*0.1226*)	-0.0406(*0.1426*)
**DW**	r_G	**0.6400**(*0.1150*)	**0.6730**(*0.1694*)	**-**
	β	**0.1820**(*0.0422*)	**0.3610**(*0.1068*)	**0.1978**(*0.0855*)
	r_{POP}	**0.5834** (*0.0942*)	**0.6586**(*0.0808*)	**0.4656**(*0.1118*)
	r_G	**-0.4260**(*0.1847*)	0.3090(*0.2634*)	-
**SGP**	β	**-0.1446**(*0.0711*)	0.2204(*0.1963*)	-0.2725(*0.1414*)
	r_{POP}	-0.1812(0.1381)	-0.0684(*0.1421*)	-0.1062(*0.1412*)

References

Greenberg CH (2000) Individual variation in acorn production by five species of southern Appalachian oaks. For Ecol Manag 132:199–210

Guyette RP, Muzika R, Kabrick J, Stambaugh MC. 2004 A perspective on Quercus life history characteristics and forest disturbance. In: Spetich M (ed) Proceedings of the upland oak ecology symposium. Gen. Tech. Rep. SRS-73. US Department of Agriculture, Forest Service, Southern Research Station, Asheville, 311 pp

Ramírez-Valiente, J.A., Etterson, J.R., Deacon, N.J., Cavender-Bares, J., 2019. Evolutionary potential varies across populations and traits in the neotropical oak *Quercus oleoides*. Tree Physiol. 39, 427–439. https://doi.org/10.1093/treephys/tpy108

Santos-del-Blanco, L., Climent, J., González-Martínez, S.C., Pannell, J.R., 2012. Genetic differentiation for size at first reproduction through male versus female functions in the widespread Mediterranean tree *Pinus pinaster*. Ann. Bot. 110, 1449–1460. https://doi.org/10.1093/aob/mcs210
