# Supplementary material for: Adaptive potential of maritime pine under contrasting environments
Source: BMC Plant Biol. 2024 Jan 9;24:37. doi: 10.1186/s12870-023-04687-w (PMC10775667; doi:10.1186/s12870-023-04687-w)
Supplement: Supplementary file 2 — Additional file 2. [file 12870_2023_4687_MOESM2_ESM.pdf]

Methods S2 Evaluation of $\delta^{13}C$ and Phenology growth index. Methods for $\delta^{13}C$ determination and variation among years and sites.

Carbon isotope composition ($\delta^{13}C$), a measure of Water Use Efficiency, was measured in two consecutive years. Measurements were made in mid-August in two consecutive years, $\delta^{13}C\_5$, $\delta^{13}C\_6$, corresponding to the 5th to the 6th growing year after seeds respectively, with contrasting water availability (a reduction greater than 35% of summer rainfall among the two consecutive years, and a slightly increment of 12% in mean temperature). A bulk of 5 needles positioned 10 cm below the upper part of the shoot to avoid sampling bias (Warren et al., 2001) were sampled and prepared in a standard way for analysis (Brendel, 2001). Determination of $\delta^{13}C$ was performed with a mass spectrometer at the University of Colorado isotope laboratory. Raw values were corrected by their position in the plate according to the standards, and this value was used for the subsequent analysis.

The differences in climate correspond to the values of the isotopic discrimination in the two sites (Figure S2.1), and in the main statistics of isotopic discrimination (Table S2.1).

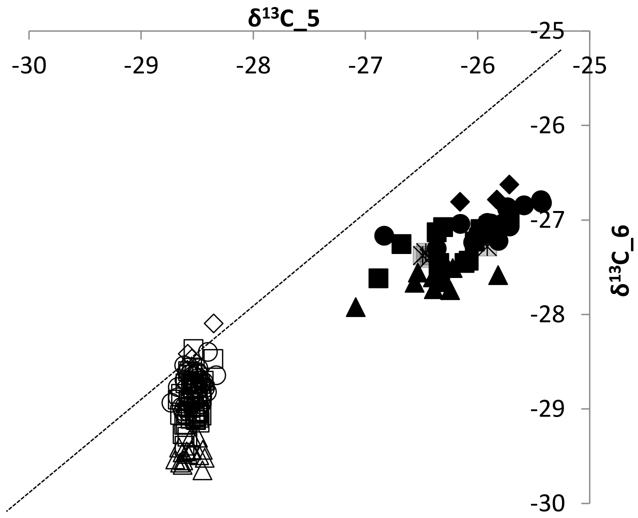

Figure S2.1. Phenotypic value of the families for Carbon isotopic composition in the two years and for the two sites (not filled black symbols: *HiProd* site, filled black symbols: *LoProd Site*; Genetic Groups of Maritime pine are French Atlantic (□); Iberian Atlantic (o); Central Spain (▲), Southern Spain(◊) and Morocco (☒)). The line of no phenotypic plasticity is included.

Table S2.1. Values of Carbon isotopic composition ($\delta^{13}C$) for two consecutive years in two contrasting environments.

Site	Trait	Mean	std	Q_{ST}	std	h^2	std
HiProd	$\delta^{13}C$ _5	-28.37	0.076	ne	ne	0.317	0.096
	$\delta^{13}C$ _6	-27.38	0.304	0.114	0.0522	0.536	0.12
LoProd	$\delta^{13}C$ _5	-28.76	0.370	0.047	0.002	0.63	0.129
	$\delta^{13}C$ _6	-28.06	0.300	0.214	0.005	0.304	0.095

Phenology growth index

Annual shoot growth can be divided in components with different importance among families or populations in Pines. Perry et al. (1966) determined that the annual growth rate is responsible of the 60% of the variation in the total growth and this variation is shown among families (Magnussen and Yeatman, 1989). Also, Jayawickrama et al. (1998) showed that some differences among families were related to the duration of the growing period. Both duration and growth rate present high degree of genetic control (Rweyongeza et al., 2004; Notivol et al., 2007).

Annual shoot growth in pines can be described (Notivol, 2020) following a Gompertz function:

$$H = ce^{-e^{-b(t-m)}}$$

where H is the height of the plant (in mm) at day t (julian days), c is the total height at the end of the growing season (asymptote), b is related to the ratio of the maximum daily growth to the total height (b≈crecimientomax/c), and m is the t corresponding to the inflection point of the curve (day of maximum growth rate).

Figure S2.2 represent the variation in some of the different parameters of the annual growth curve.

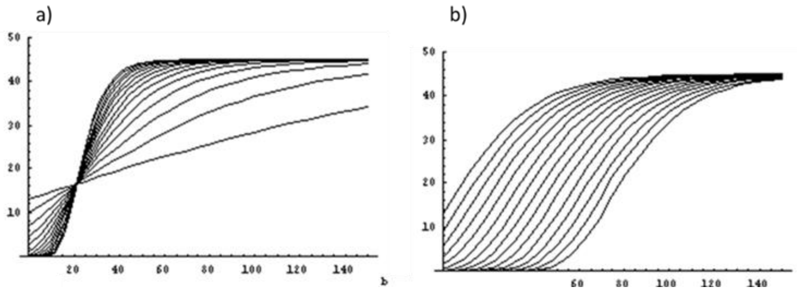

Figure 2.2. a) variation in the b parameter, and b) in the m parameter of a growth Gompertz funcion (Notivol, 2020).

In relation to the m parameter, Figure 2.3 represent the value of the Phenology growth index.

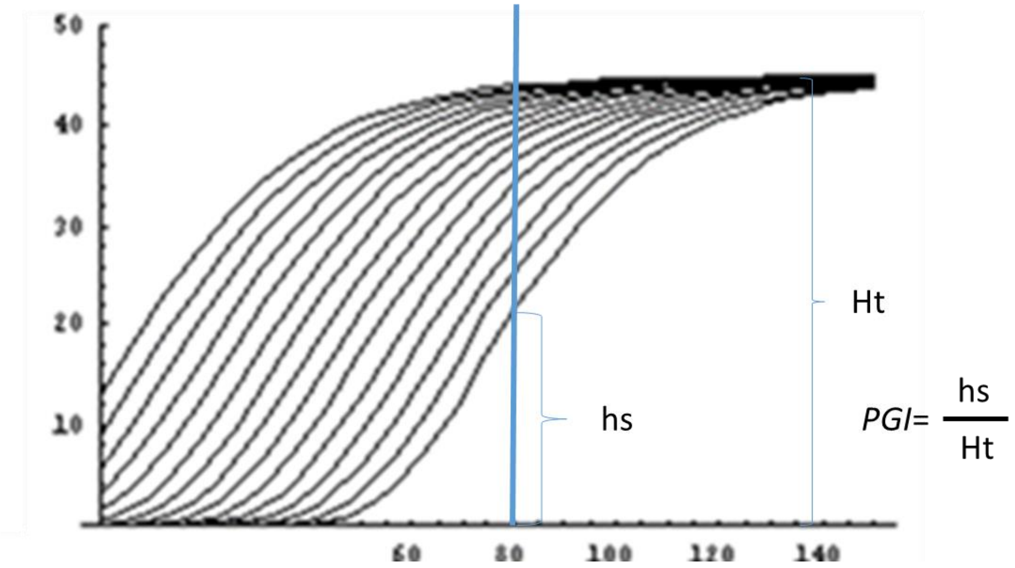

Figure 2.2. Phenology growth index measured for different Gompertz functions differing in the m parameter (Notivol 2020).

In the two common garden, we estimated the gompertz function by measuring height along the growing season. We derived different variables of the curve PGI (phenology growth index), T10 (julian day when the tree reach the 10% of the growth), T50 (julian day when the tree reach the 50% of the growth), T90 (julian day when the tree reach the 90% of the growth), duration (T90-T10). We obtained the correlation among those traits to select one trait explaining the differences in growth phenology (Table S2.2).

Table S2.2. Pearson Correlation coefficient among variables related to the growth phenology of families maritime pine in HiProd site.

	**PGI**	**t50**	**dur**	**t90**	**t10**	**m**	**b**	**c**
**PGI**	1,000	**-0,677**	-0,243	-0,333	-0,391	**-0,607**	0,018	-0,150
**t50**		1,000	0,629	0,395	0,253	**0,893**	-0,152	0,018
**dur**			1,000	**0,960**	**-0,644**	0,295	**-0,703**	0,174
**t90**				1,000	-0,402	-0,001	**-0,644**	0,240
**t10**					1,000	0,533	**0,517**	-0,077
**m**						1,000	0,116	-0,096
**b**							1,000	-0,029
**c**								1,000
	901	902	687	816	734	902	902	902

We observe that the growth phenology index is mostly related to the m parameter of the Gompertz function, i.e. the moment where the curve reach the maximum daily growth and therefore, a highest value indicates a delay in the growth phenology.

References

Brendel, O. (2001). Does bulk-needle delta C-13 reflect short-term discrimination? *Ann. For. Sci.* 58, 135–141.

Jayawickrama, K. J. S., McKeand, S. E., and Jett, J. B. (1998). Phenological variation in height and diameter growth in provenances and families of loblolly pine. *New For.* 16, 11–25.

Magnussen, S., and Yeatman, C. W. (1989). Height growth components in inter- and intra-provenance jack pine families. *Can. J. For. Res.* 19, 962–972.

Notivol, E. 2020. Mejora genetica de Pinus sylvestris L.: Estudio de procedencias y estrategias de conservacion y mejora genetica. Tesis Doctoral. UPM. Madrid. Spain

Notivol, E., Garcia-Gil, M. R., Alía, R., and Savolainen, O. (2007). Genetic variation of growth rhythm traits in the limits of a latitudinal cline in Scots pine. *Can. J. For. Res.* 37, 540–551. doi:10.1139/X06-243.

Perry, T., Chi-Wu, W., and Schmitt, D. (1966). Height Growth for Loblolly Pine provenances in relation to photoperiod and growing season. *Silvae Genet*.

Rweyongeza, D. M., Yeh, F. C., and Dhir, N. K. (2004). Genetic parameters for seasonal height and height growth curves of white spruce seedlings and their implications to early selection. *For. Ecol. Manage.* 187, 159–172.

Warren, C., McGrath, J., and Adams, M. (2001). Water availability and carbon isotope discrimination in conifers. *Oecologia* 127, 476–486. doi:10.1007/s004420000609.
