# Supplementary material for: Adaptive potential of maritime pine under contrasting environments
Source: BMC Plant Biol. 2024 Jan 9;24:37. doi: 10.1186/s12870-023-04687-w (PMC10775667; doi:10.1186/s12870-023-04687-w)
Supplement: Supplementary file 3 — Additional file 3. [file 12870_2023_4687_MOESM3_ESM.pdf]

Methods S3 Climatic characterization of *Pinus pinaster* populations and sites.

Table S3.1. Characteristics of the populations of *Pinus pinaster* Ait. and the two common gardens.

					Climatic data[1]				
**Location**	**Code**	**Altitude**	**Longitude**	**Latitude**	**MAT**	**MTWM**	**MTCM**	**AP**	**SP**
Mimizan-FR	FA1	37	44.13417N	-1.30317W	13.28	24.80	3.20	1235	232
Pleucadec-FR	FA2	80	47.78119N	-2.34367W	11.24	21.90	2.50	804	154
Cadavedo-SP	IA1	210	43.53996N	-6.41785W	13.22	22.00	5.01	1316	204
Puerto de Vega-SP	IA2	121	43.54795N	-6.63137W	13.39	22.61	4.91	1283	194
Leiria-PT	IA3	20	39.78333N	-8.95750W	15.35	24.40	7.40	811	44
Arenas de San Pedro-SP	CS1	733	40.19482N	-5.11621W	14.18	33.41	1.24	1318	73
Coca-SP	CS2	800	41.25470N	-4.49782W	12.28	31.18	-0.57	454	77
Cuéllar-SP	CS3	830	41.37460N	-4.48403W	12.17	30.93	-0.65	468	72
Bayubas-SP	CS4	998	41.52297N	-2.87743W	10.63	29.59	-1.36	553	96
Oria-SP	SS1	1223	37.53116N	-2.35113W	13.14	30.70	0.44	357	29
Tamrabta-MO	MO1	1758	33.60000N	-5.01666W	10.66	30.40	-4.60	745	49
Cabada- SP	*HiProd*[2]	455	43.41947N	-6.53966W	12.92	23.58	3.69	1316	134
Ibias-SP	*LoProd*[3]	738	43.02157N	-6.88400W	12.94	28.22	2.17	1754	142

[1] MAT-mean annual temperature, MTWM-mean of maximum temperature of the warmest month, MTCM-mean of minimum temperature of the coldest month, AP-total annual precipitation and SP-summer precipitation- . Site climatic data is the average for the period from planting to field measurements. [2]Site index: 22 m at 20 years, northwest orientation, 75 cm average soil depth of, Lusitanean environmental zone (Metzger, 2018). [3]Site Index: 6 m at 20 years. South orientation, 20 cm average soil depth, Mediterranean Mountain environmental zone.

Climatic represetantion of sampling material and common garden experiments.

We used information from 53 natural populations of maritime pine covering the distribution range of the species and the planting sites, including the two sampling years (see table S3.2).

Table S3.2. Code of the populations analysed for the climatic characterization.

Code	POPULATION	Region	Country	Code	POPULATION	Region	Country
1	Hourtin	Landas	FR	28	Bayubas de Abajo	Soria	ESP
2	Leverdon	Landas	FR	29	San Leonardo	Soria	ESP
3	Mimizan	Landas	FR	30	Boniches	Cuenca	ESP
4	Olonne sur Mer	West of France	FR	31	Olba	Teruel	ESP
5	Petrocq	Landas	FR	32	Sinarcas	Valencia	ESP
6	Pleucadec	West of France	FR	33	Sierra Calderona	Valencia	ESP
7	St-Jean des Monts	West of France	FR	34	Quatretonda	Valencia	ESP
8	Alto de la Llama	Tineo / Asturias	ESP	35	La Bisbal	Girona	ESP
9	Armayán	Tineo / Asturias	ESP	36	Cazorla	Jaén	ESP
10	Cadavedo	Valdés / Asturias	ESP	37	Cómpeta	Málaga	ESP
11	Sierra de Barcia	Valdés / Asturias	ESP	38	Oria	Almería	ESP
12	Castropol	Asturias	ESP	39	Madisouka	Rif	MOR
13	Lamuño	Asturias	ESP	40	Tamrabta	Atlas	MOR
14	Puerto de Vega	Navia / Asturias	ESP	41	Sidi-Meskour	High Atlas	MOR
15	Rodoiros	Asturias	ESP	42	Tabarka	Tunisia	TU
16	Sergude	A Coruña	ESP	43	Ahín	Castellón	ESP
17	S. Cip. Ribaterme	Pontevedra	ESP	44	Almazan	Soria	ESP
18	Leiria	Portugal	PT	45	Aullene	Francia	FR
19	Pineta	Córcega	FR	46	Benicassim	Castellón	ESP
20	Pinia	Córcega	FR	47	El Sahúgo	Salamanca	ESP
21	Tabuyo del Monte	León	ESP	48	Erdeven	Francia	FR
22	Arenas de San Pedro	Ávila	ESP	49	Fuencaliente	C Real	ESP
23	Valdemaqueda	Madrid	ESP	50	Fuentelapeña	Zamora	ESP
24	Cenicientos	Madrid	ESP	51	Gaucín	Málaga	ESP
25	Coca	Segovia	ESP	52	Oña	Burgos	ESP
26	Cuellar	Segovia	ESP	53	Sierra del Pradell	Tarragona	ESP
27	Carbonero el Mayor	Segovia	ESP				

Based on 5 climatic variables, we obtained a PCA, where the two first components explained 54.7% of the total variation (Table S3.3).

Table S3.3. Variance explained by each principal component for 58 populations of *Pinus pinaster* Aiton.

	Comp.1	Comp.2	Comp.3	Comp.4
Standard deviation	1.5727484	1.3271058	0.7127598	0.46707159
Proportion of Variance	0.4947075	0.3522420	0.1016053	0.04363117
Cumulative Proportion	0.4947075	0.8469495	0.9485548	0.99218597

The biplot of variables and observations is presented in figure S3.1.

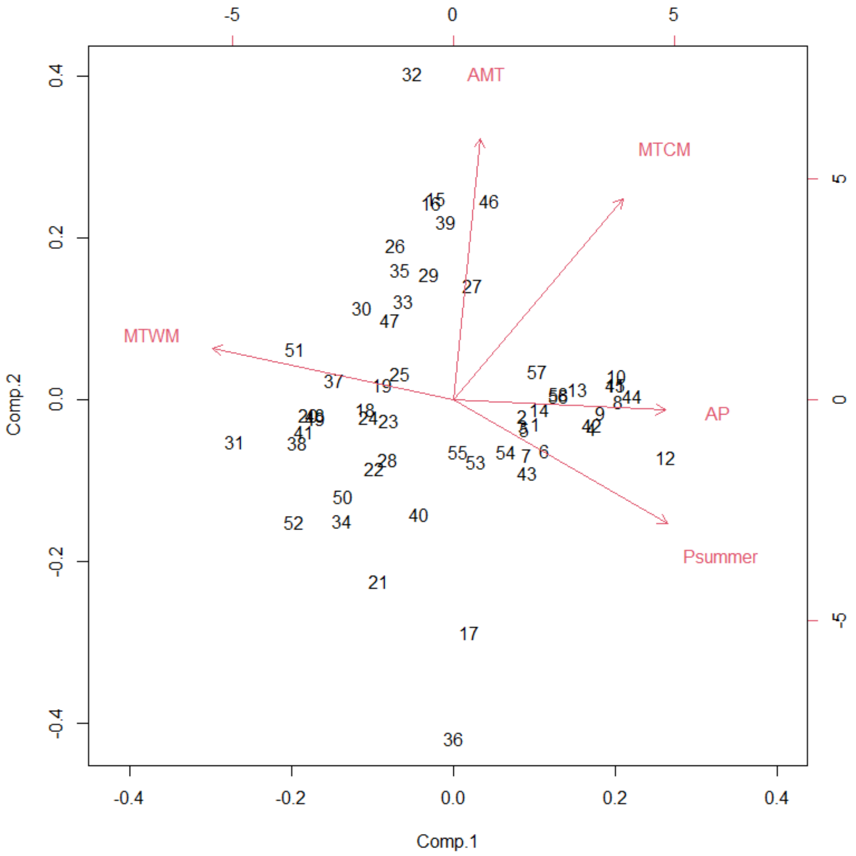

Figure S3.1. Biplot of variables and observations of populations and common gardens used in the study. (Codes as in Table S.3.2)

The sampled populations covered the distribution range of the species, and the two sites and the sampling years differed (Figure S3.2).

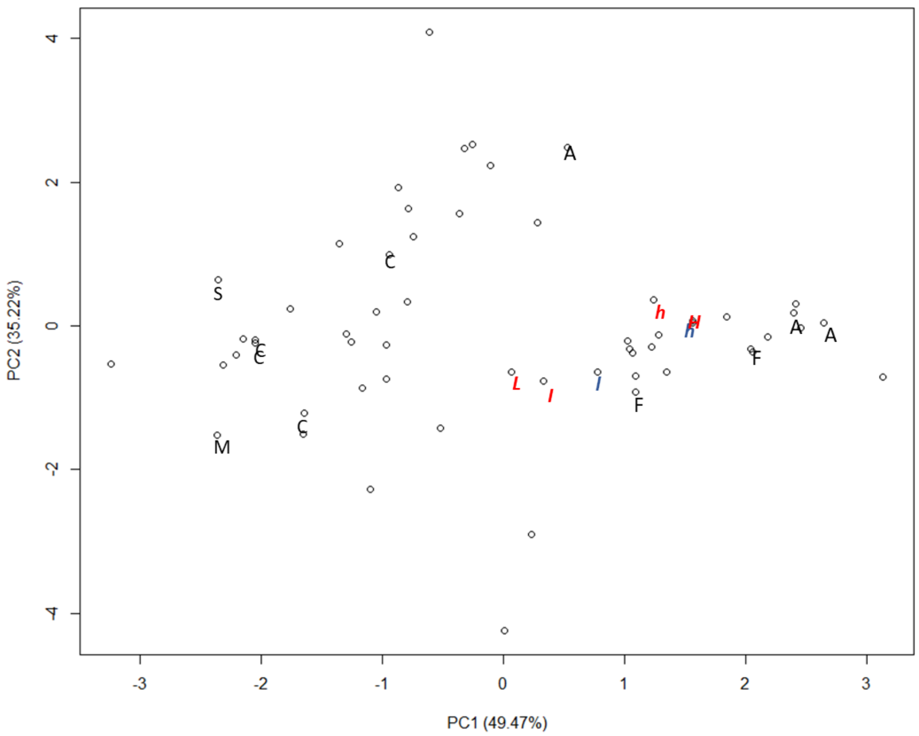

Figure S3.2. Plot of the two fist PC of maritime pine populations (A: Atlantic, F: French, C: Central Spain, S: Shouthern Spain, M: Morocco) and sites (***H***: mean valued of Hiprod site, *h*: values 5th year Hiprod, *h*: value 6th year Hiprod site; ***L***: mean valued of Loprod site, *l*: values 5th year Loprod, *l*: value 6th year Loprod site). (Points represent the populations from table S.3.2)

Ombrothermic diagram of the two sites

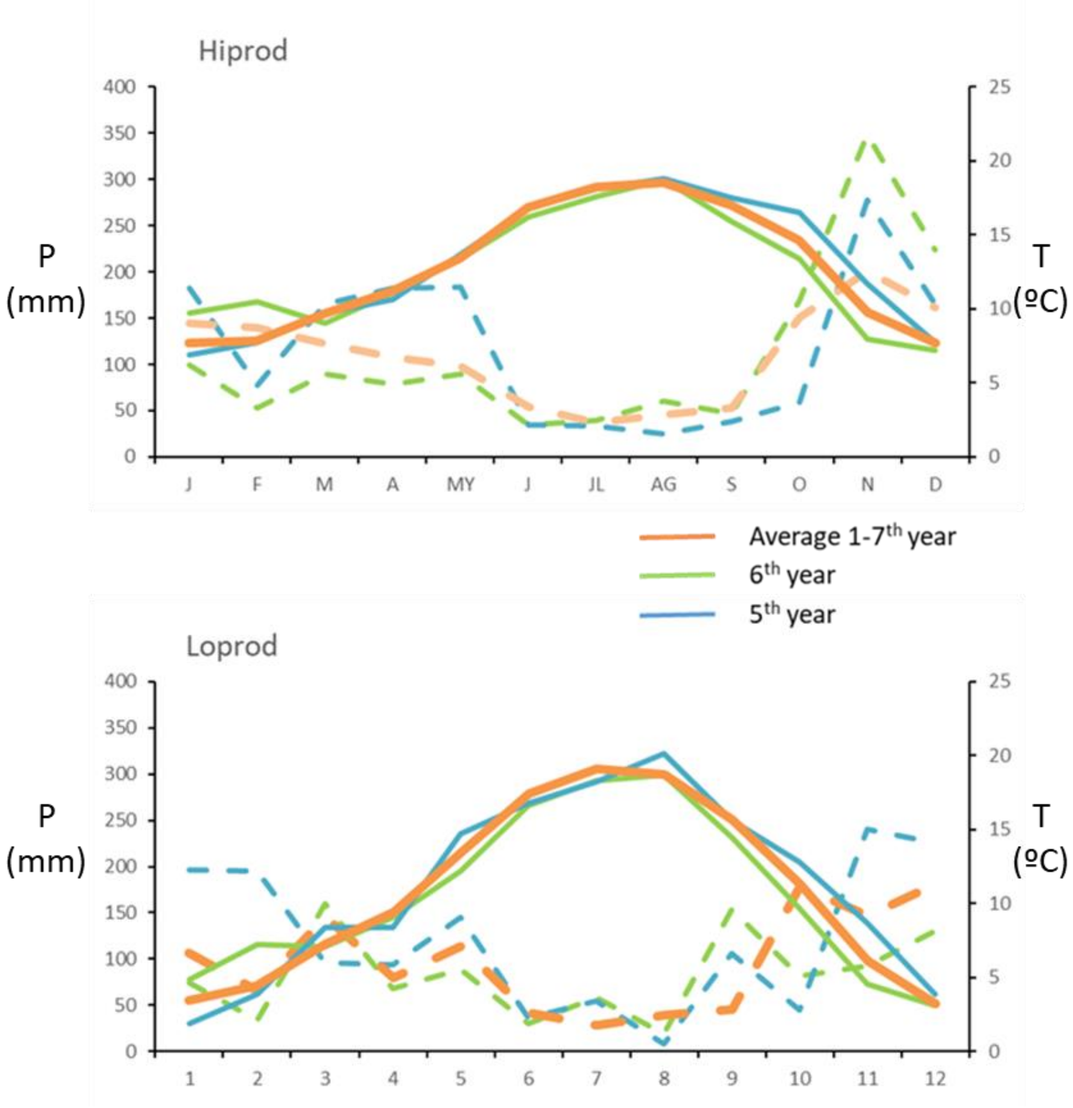

Figure S3.3. Ombrothermic diagrams of the two sites (P, T: monthly precipitation and temperatures). The values corresponding to the average (1-7th year) and the two years were the D13C were measured (5th and 6th).
