# Supplementary material for: Adaptive potential of maritime pine under contrasting environments
Source: BMC Plant Biol. 2024 Jan 9;24:37. doi: 10.1186/s12870-023-04687-w (PMC10775667; doi:10.1186/s12870-023-04687-w)
Supplement: Supplementary file 4 — Additional file 4. [file 12870_2023_4687_MOESM4_ESM.pdf]

Methods S4 Statistical models.

We have checked the residuals of the models by means of the DHPlottinARMa R package (https://cran.r-project.org/web/packages/DHARMa/vignettes/DHARMa.html)

Mixed models and BLUPS estimates

a) Dispersion of the residuals, for the variables considered in the analysis

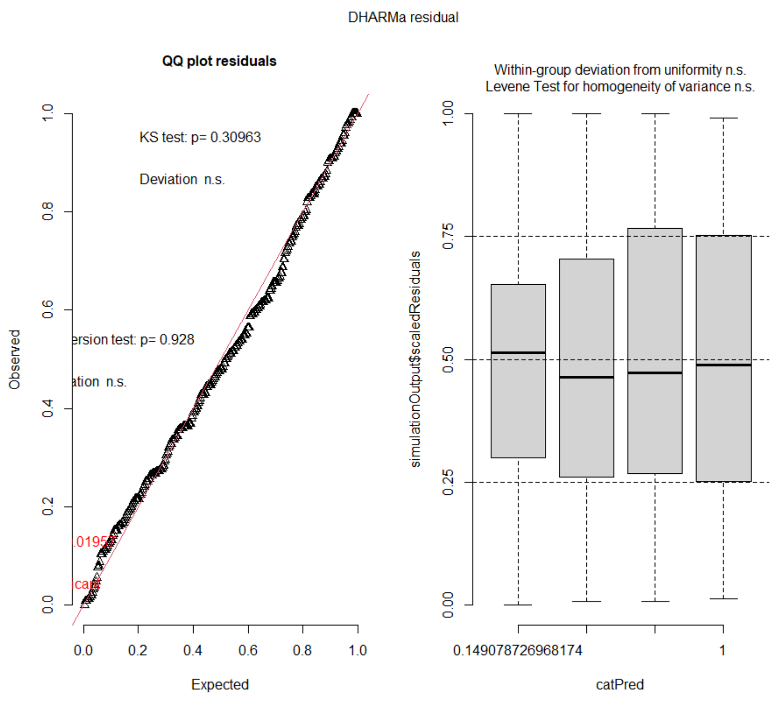

Figure S4.1 Analysis of residuals of the mixed model in the *LoProd* site- M_D13C.

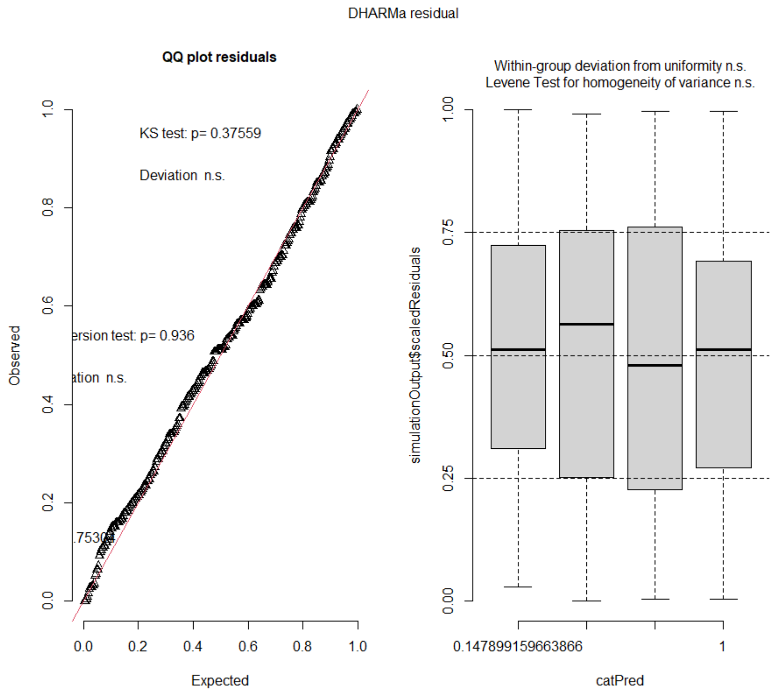

Figure S4.2 Analysis of residuals of the mixed model in the *LoProd* site- PI_D13C.

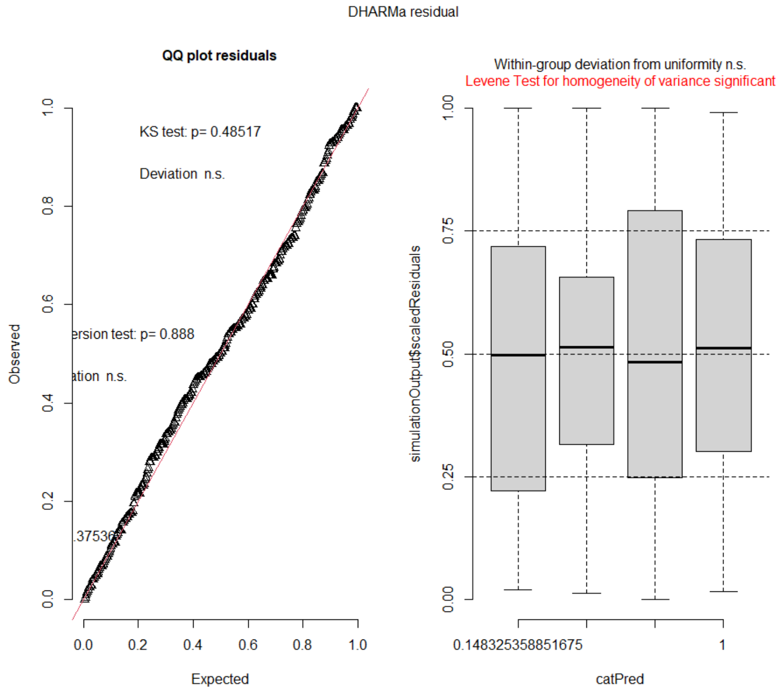

Figure S4.3 Analysis of residuals of the mixed model in the *LoProd* site- SLA.

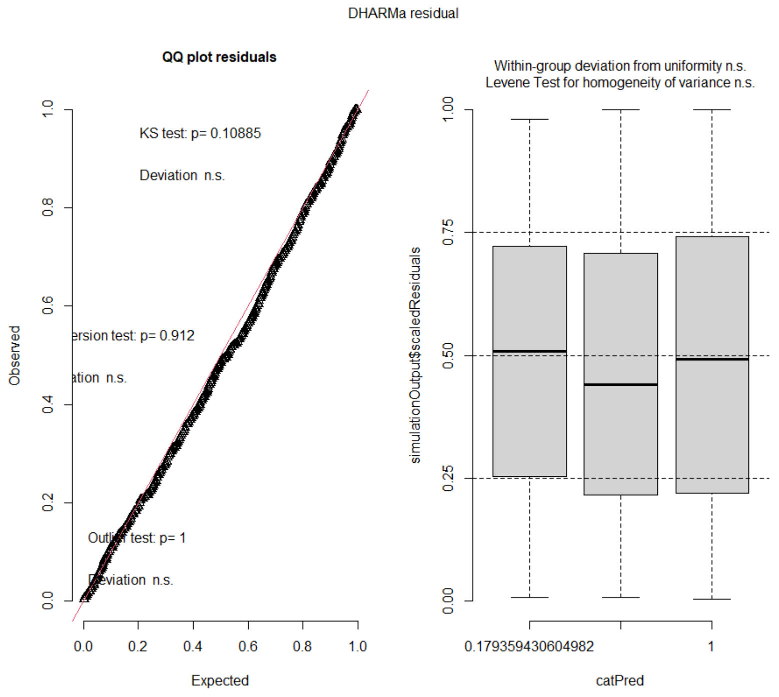

Figure S4.4 Analysis of residuals of the mixed model in the *LoProd* site- DW.

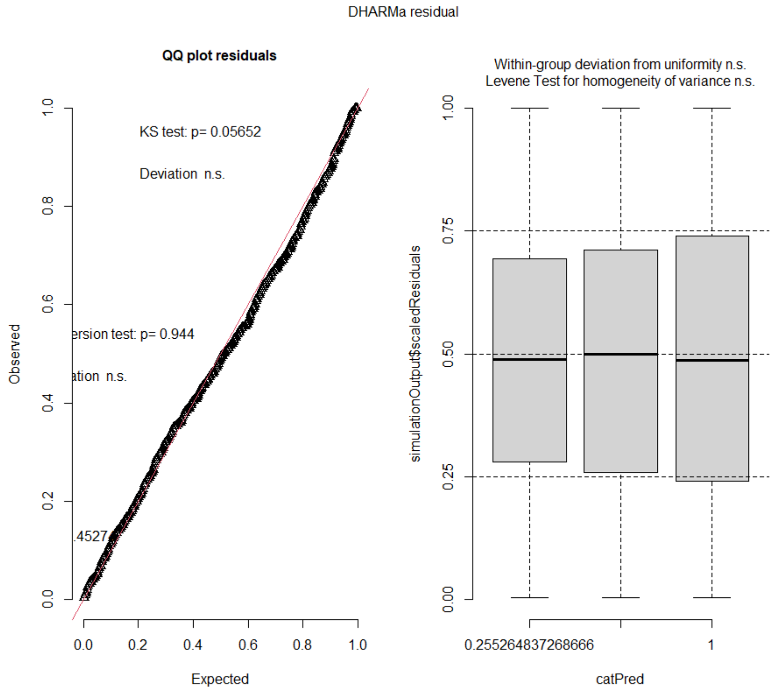

Figure S4.5 Analysis of residuals of the mixed model in the *LoProd* site- PGI.

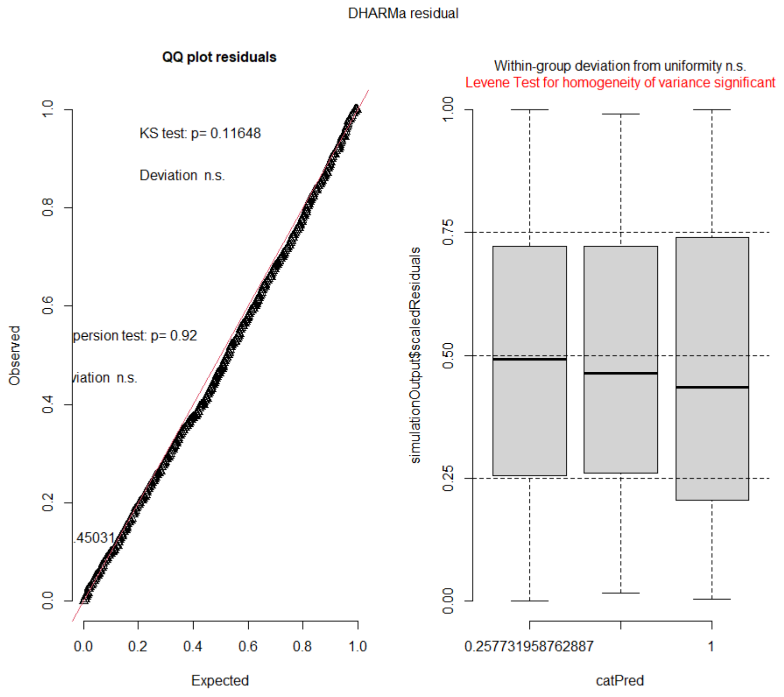

Figure S4.6 Analysis of residuals of the mixed model in the *HiProd* site- M_D13C.

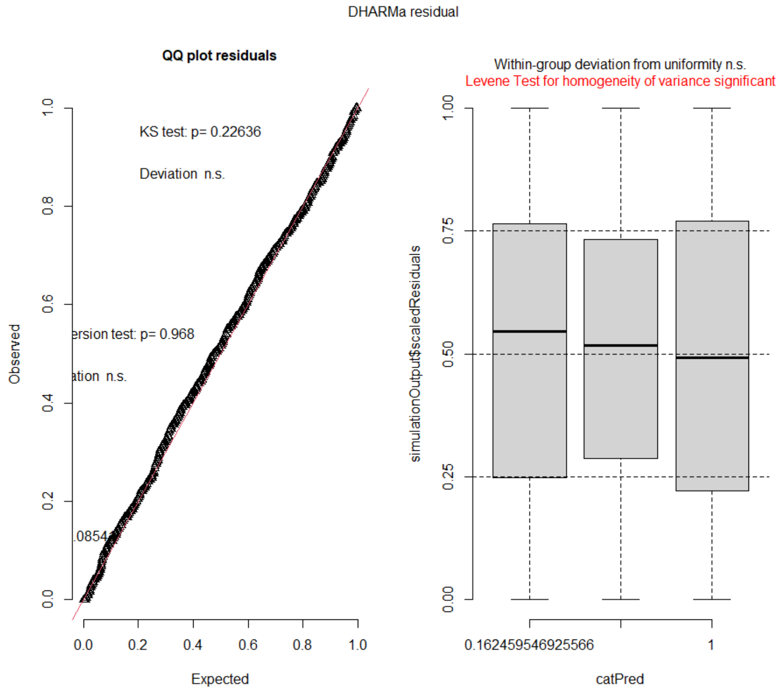

Figure S4.7 Analysis of residuals of the mixed model in the *HiProd* site- PI_D13C.

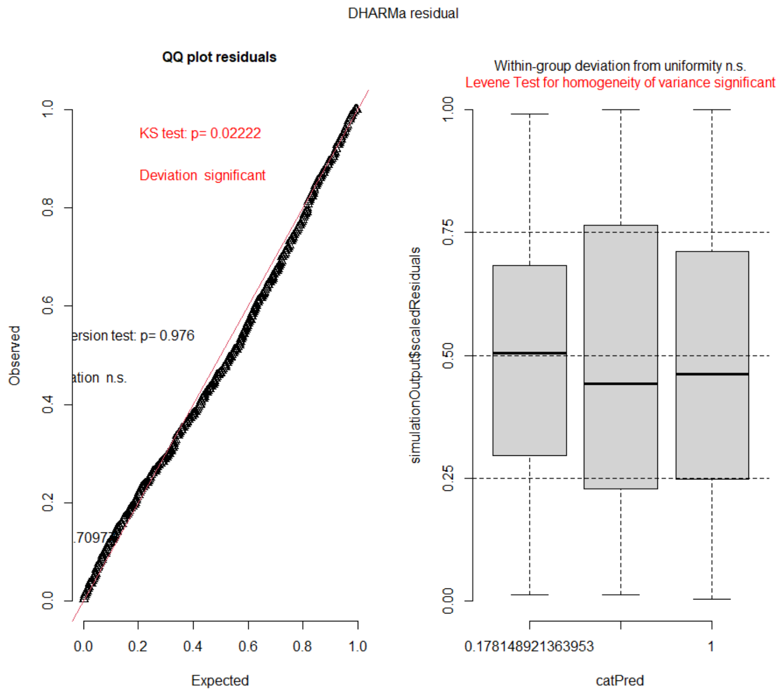

Figure S4.8 Analysis of residuals of the mixed model in the *HiProd* site- SLA.

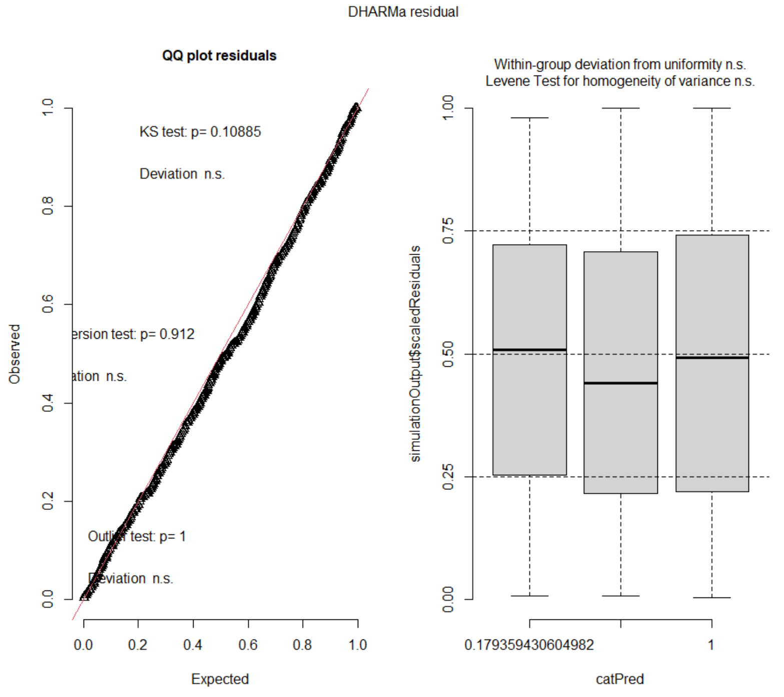

Figure S4.9 Analysis of residuals of the mixed model in the *HiProd* site- DW.

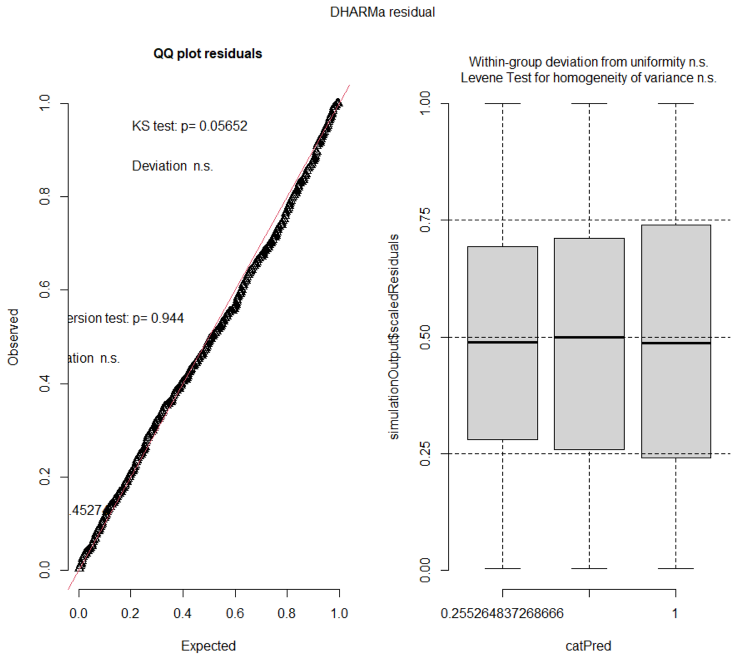

Figure S4.10 Analysis of residuals of the mixed model in the *HiProd* site- PGI.

b) Distribution of Random effects. BLUPS of families within populations.

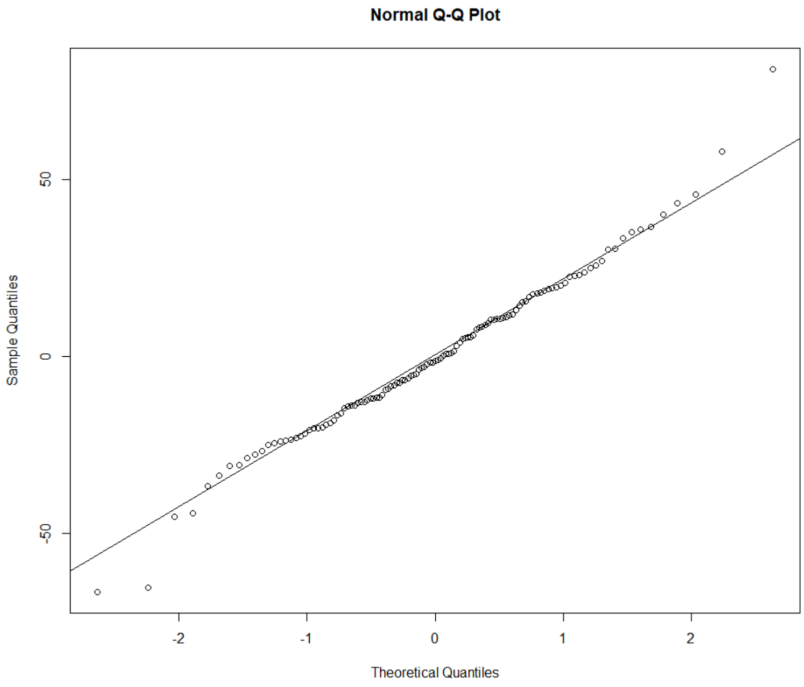

Figure S4.11 QQ plot of BLUPs in the *HiProd* site- HT.

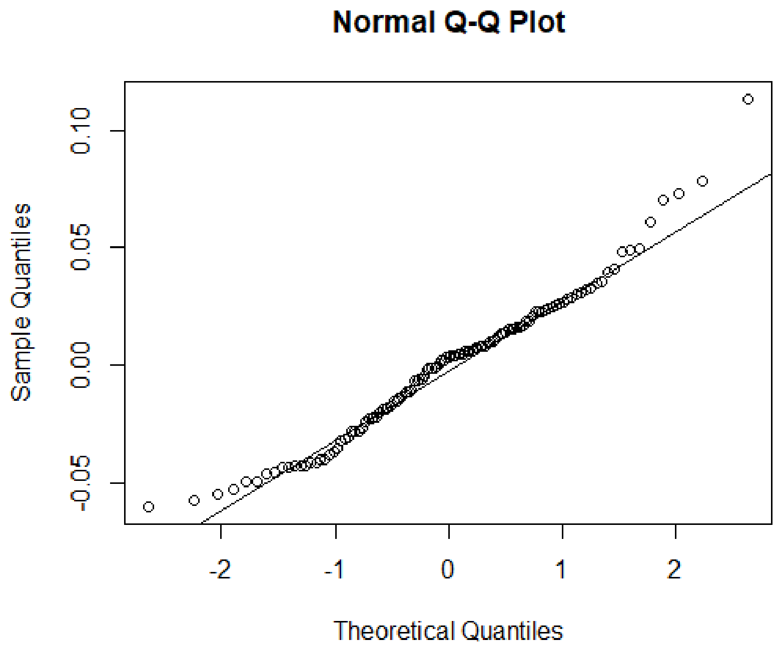

Figure S4.12 QQ plot of BLUPs in the *HiProd* site- M_D13C.

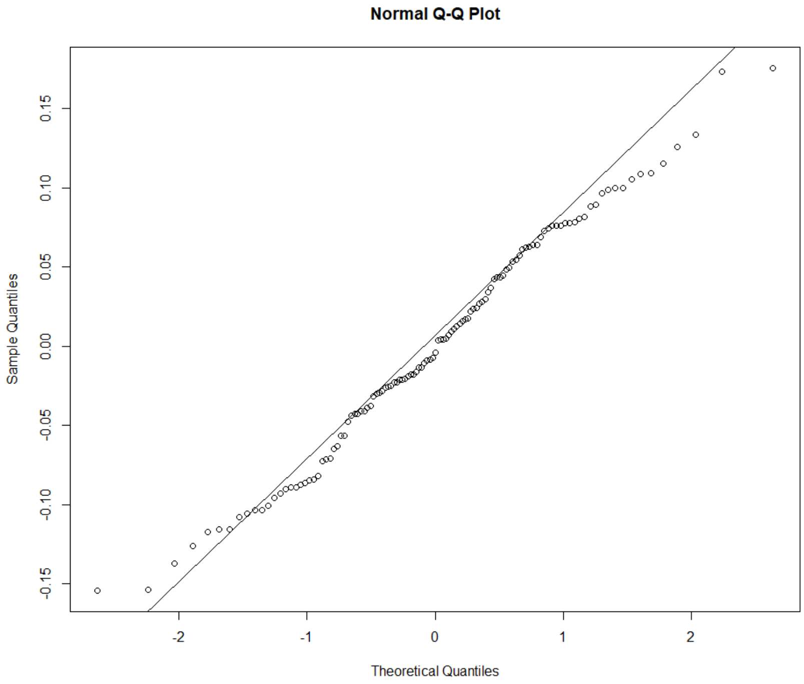

Figure S4.13 QQ plot of BLUPs in the *HiProd* site- PI_D13C.

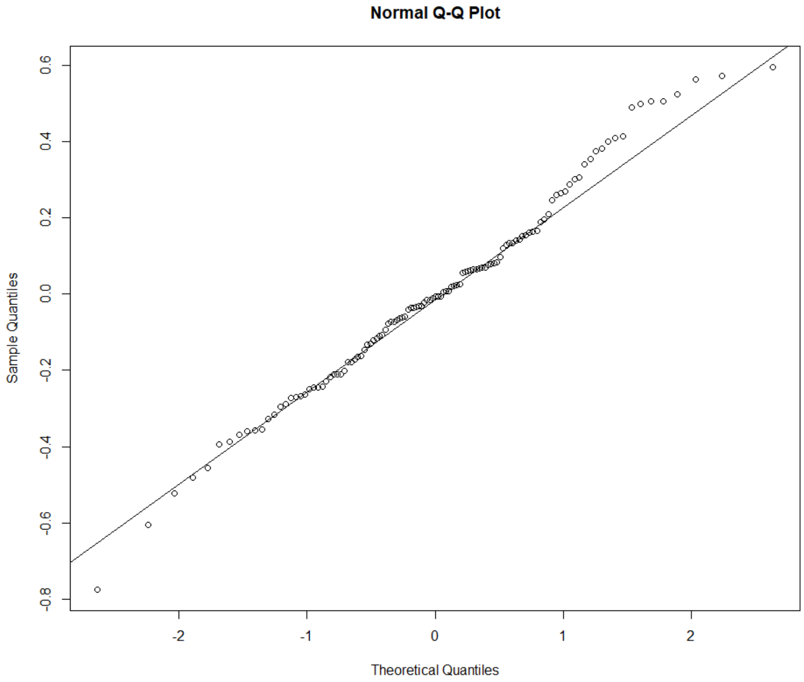

Figure S4.14 QQ plot of BLUPs in the *HiProd* site- SLA.

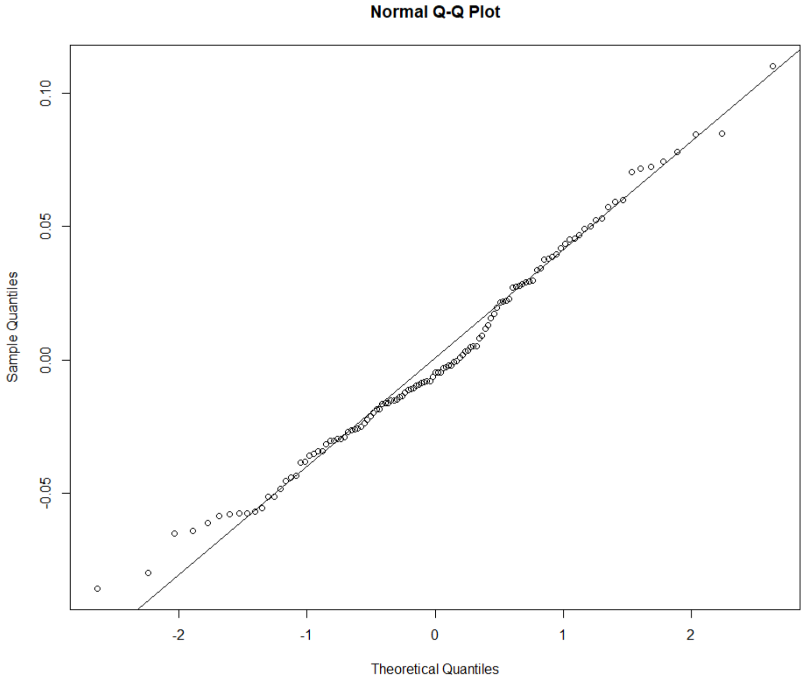

Figure S4.15 QQ plot of BLUPs in the *HiProd* site- DW.

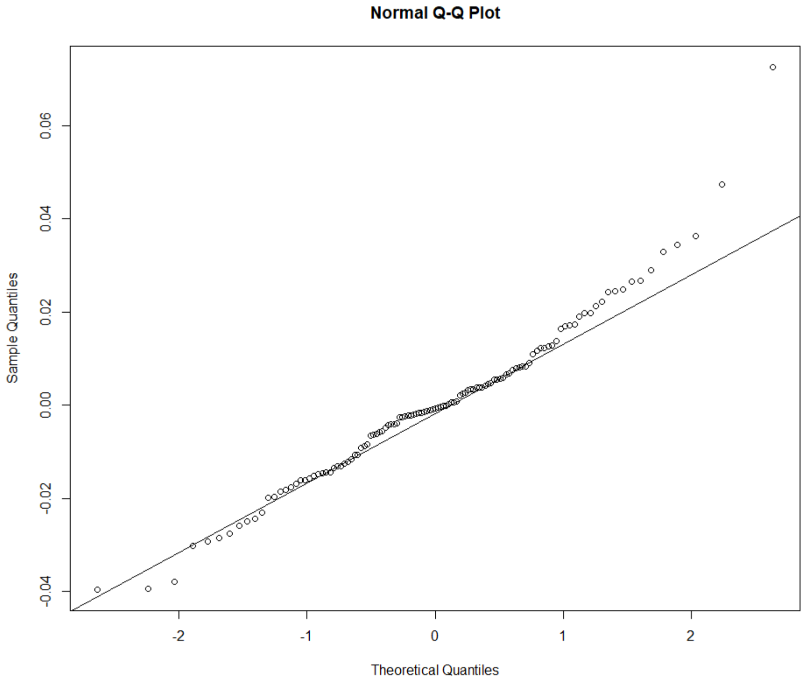

Figure S4.16 QQ plot of BLUPs in the *HiProd* site-PGI.

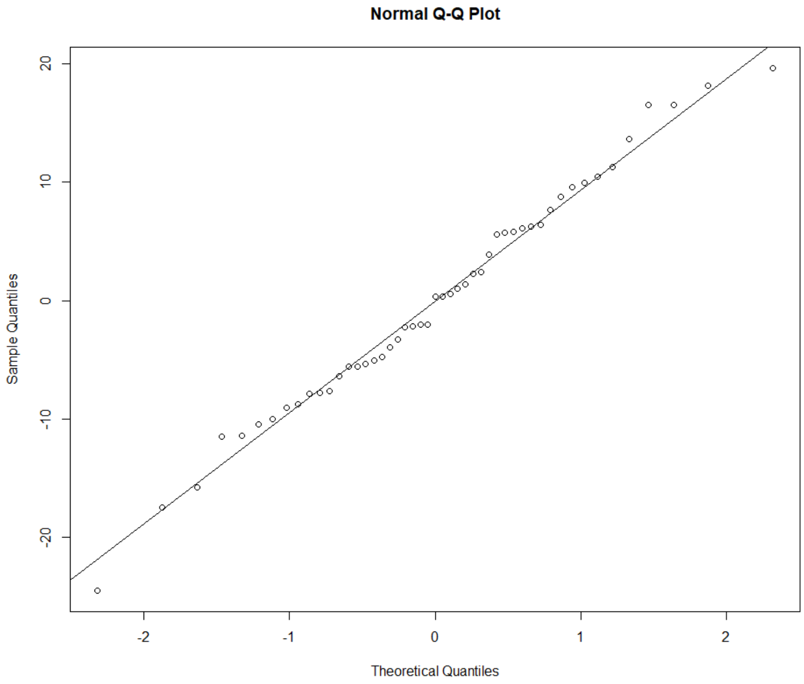

Figure S4.17 QQ plot of BLUPs in the *LoProd* site-HT.

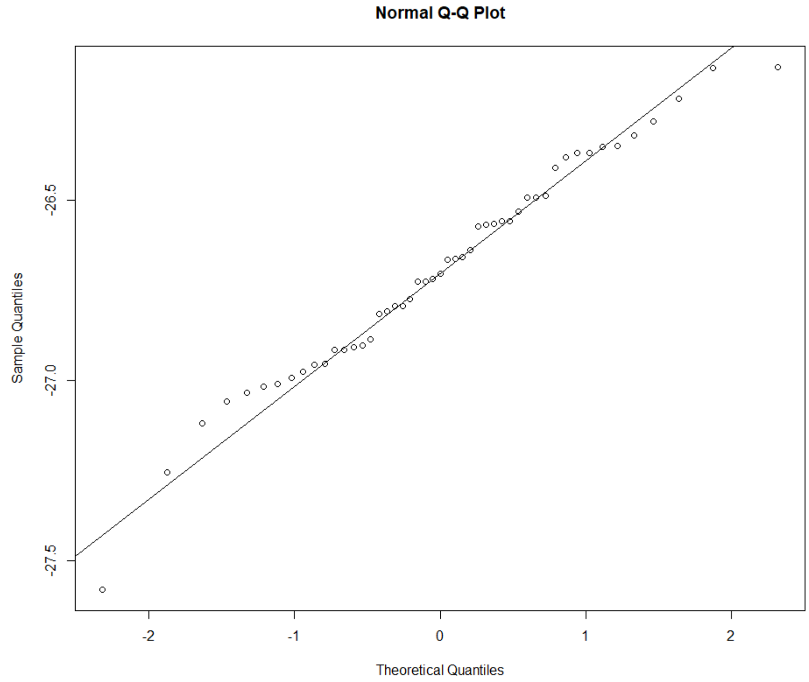

Figure S4.18 QQ plot of BLUPs in the *LoProd* site-M_D13C.

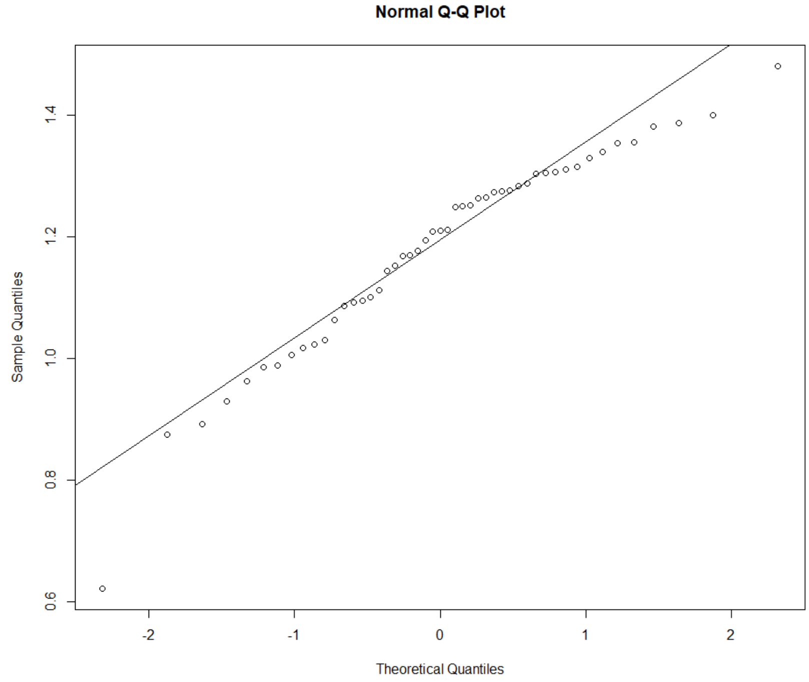

Figure S4.19 QQ plot of BLUPs in the *LoProd* site-PI_D13C.

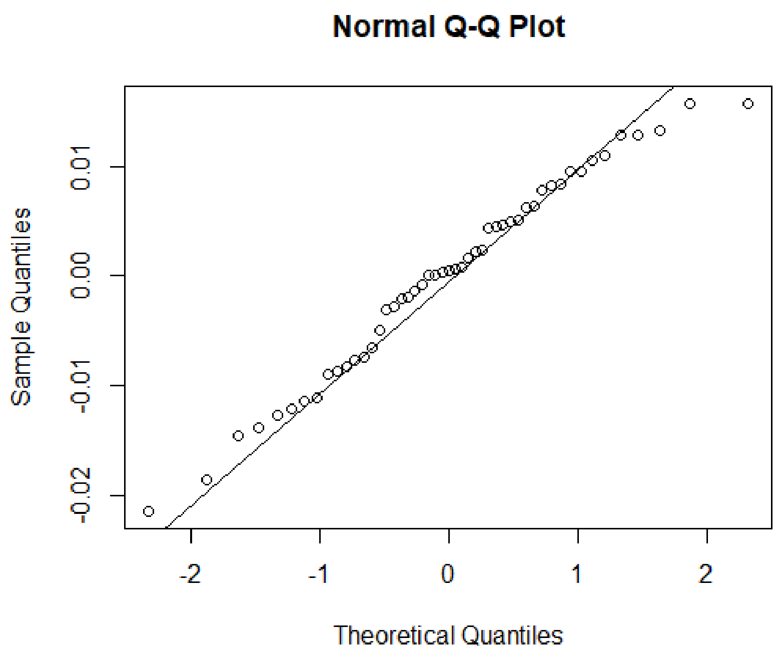

Figure S4.20 QQ plot of BLUPs in the *LoProd* site-SLA.

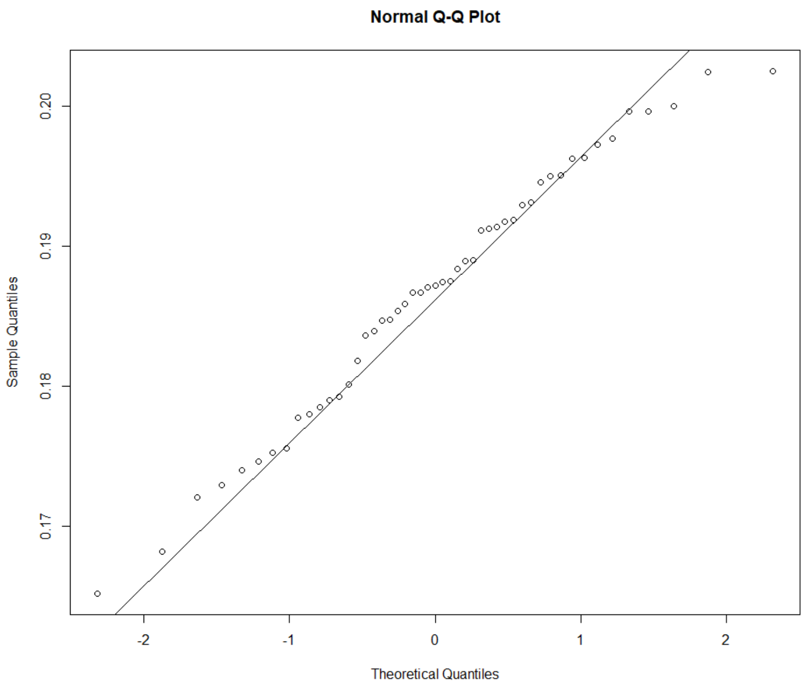

Figure S4.21 QQ plot of BLUPs in the *LoProd* site-DW.

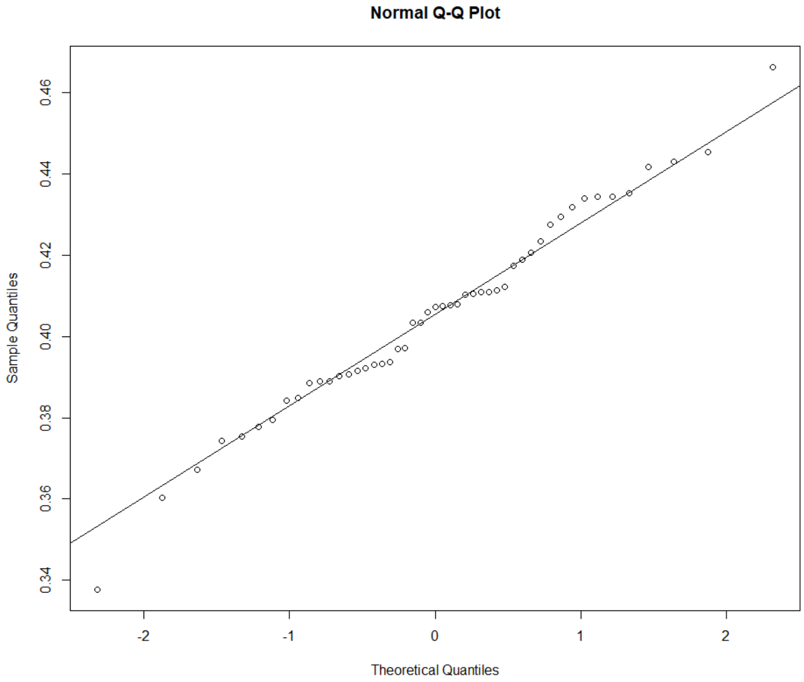

Figure S4.22 QQ plot of BLUPs in the *LoProd* site-PGI.

Log linear models

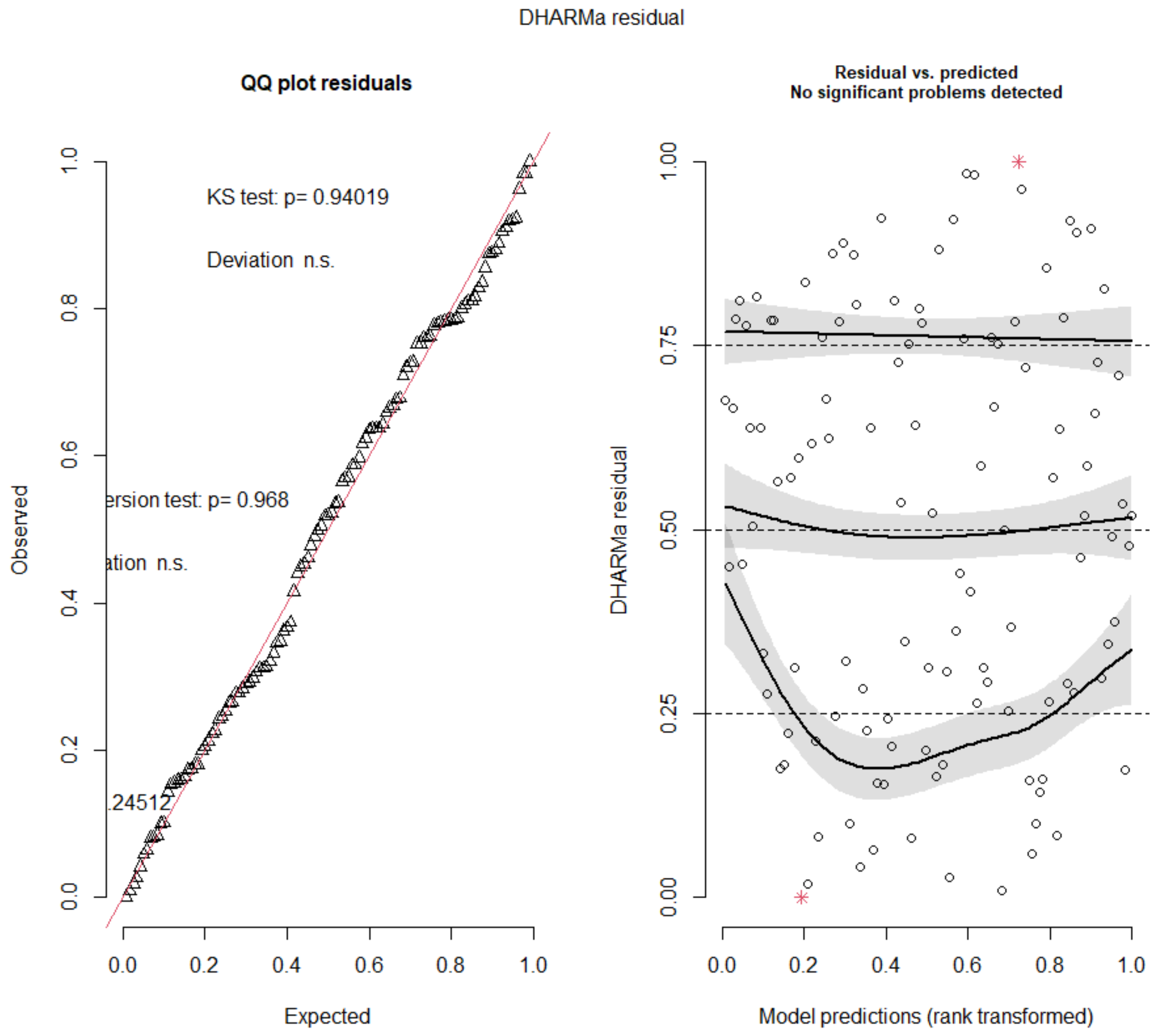

Figure S4.23 Analysis of residuals of the loglinear model in the *HiProd* site- Within population.

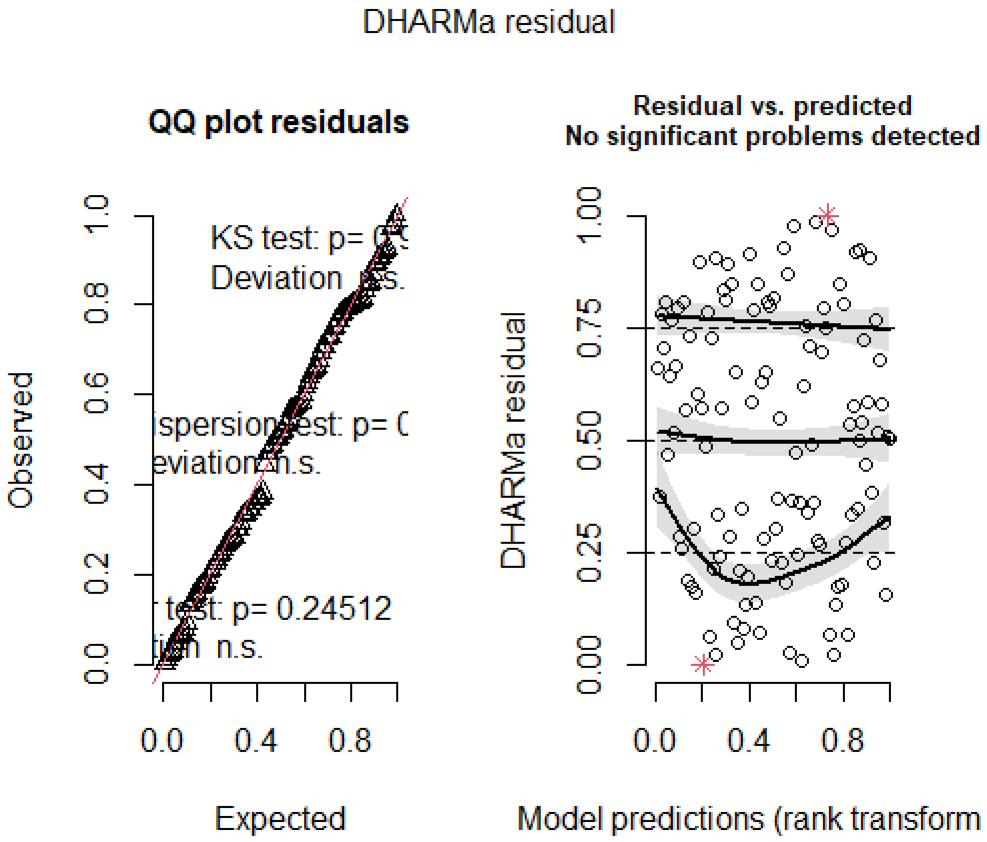

Figure S4.24. Analysis of residuals of the loglinear model in the HiProd site. Total phenotypic level

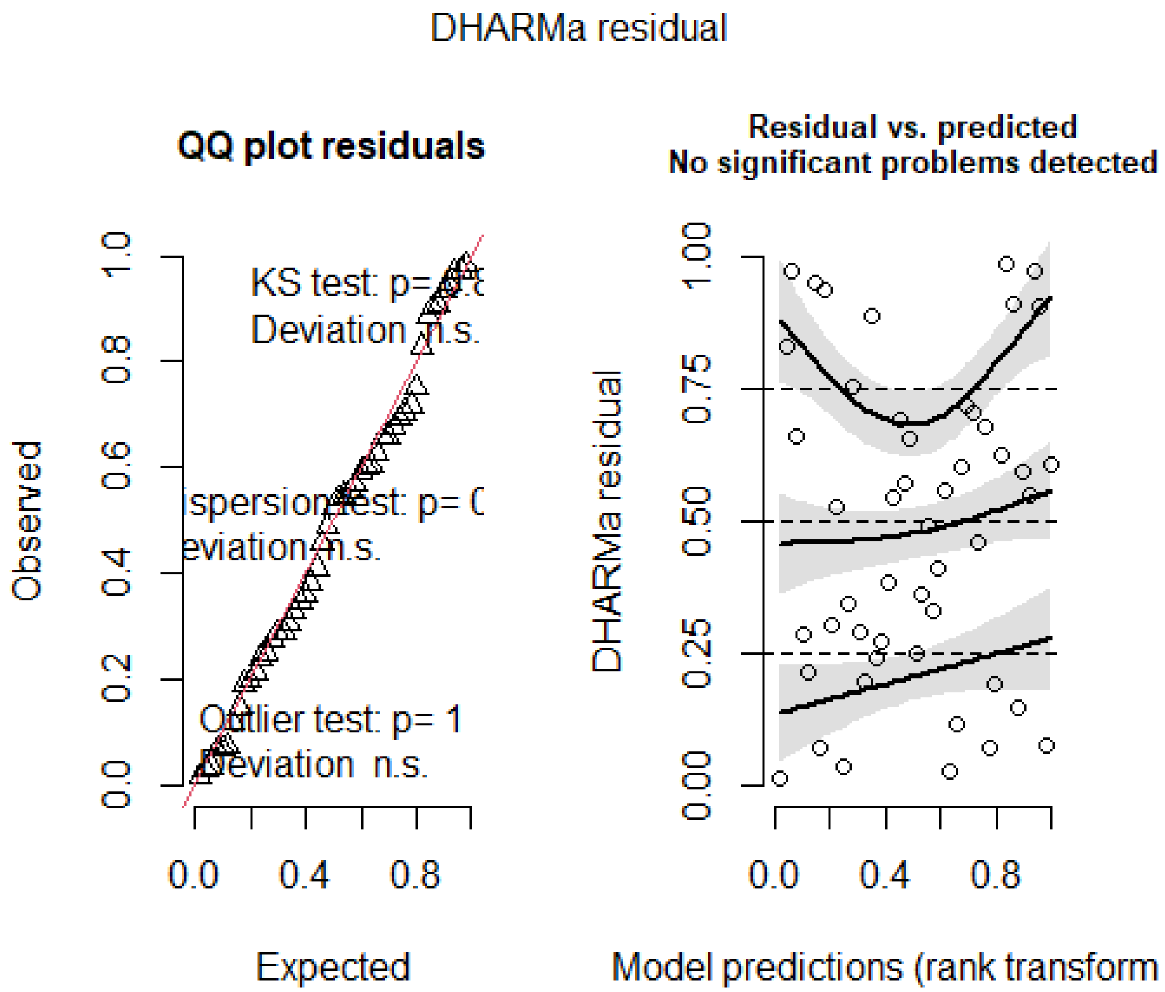

Figure S4.25 Analysis of residuals of the loglinear model in the *LoProd* site. Within population.

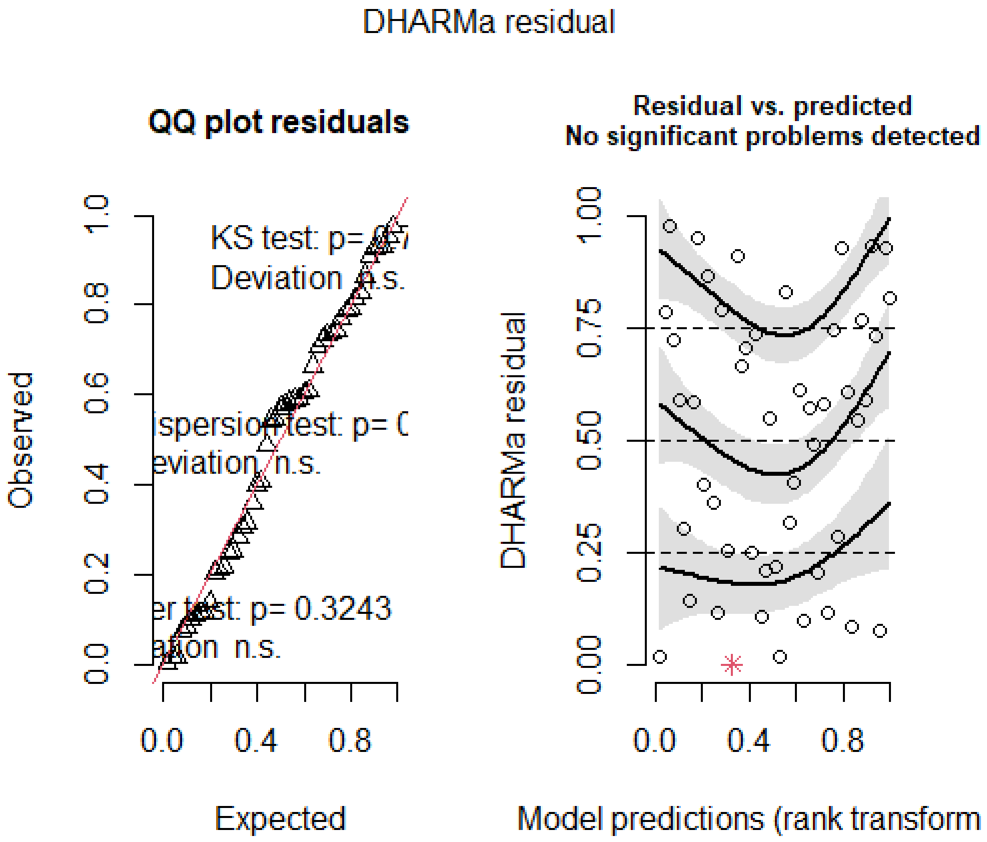

Figure S4.26 Analysis of residuals of the loglinear model in the *LoProd* site. Total phenotypic level.
