# Supplementary material for: Adaptive potential of maritime pine under contrasting environments
Source: BMC Plant Biol. 2024 Jan 9;24:37. doi: 10.1186/s12870-023-04687-w (PMC10775667; doi:10.1186/s12870-023-04687-w)
Supplement: Supplementary file 5 — Additional file 5. [file 12870_2023_4687_MOESM5_ESM.pdf]

Table S1. Mean, coefficient of genetic differentiation (Q_{ST}) heritability (h^2) and evolvability (CV_A) of fitness related traits in a Maritime pine genetic test evaluated in two sites. In brackets, standard error of estimates.

	***HiProd* Site**[1]				***LoProd* site**			
**Trait**[2]	**Mean**	**Q_{ST}**	**h^2**	**CV_A**	**Mean**	**Q_{ST}**	**h^2**	**CV_A**
**HT**	318.9	0.131 (0.019)	0.480 (0.063)	9.8	110.3	0.037 (0.043)	0.477 (0.300)	13.3
**M_D13C**	-28.56	0.064 (0.040)	0.412 (0.138)	0.7	-27.74	0.082 (0.076)	0.396 (0.240)	1.1
**PI_D13C**	0.391	0.163 (0.128)	0.163 (0.128)	41.8	0.680	0.108 (0.133)	0.248 (0.258)	26.3
**SLA**	43.19	0.069 (0.001)	0.287 (0.024)	2.8	46.21	0.334 (0.390)	0.096 (0.152)	-
**DW**	0.565	0.072 (0.017)	0.419 (0.064)	9.7	0.243	0.109 (0.056)	0.203 (0.089)	8.7
**SGP**	0.325	0.036 (0.034)	0.335 (0.213)	9.3	0.342	0.006 (0.034)	0.339 (0.067)	8.9

[1]HT: Total height at age 7; M_D13C: mean isotopic discrimination; PI_D13C: Isotopic discrimination Plasticity index, SLA: Specific leaf area; DW: Needle dry weight; SGP: Shoot growth phenology

[2]*HiProd*: High productivity site (Site index 22); *LoProd*: Low productivity site (Site index 6).
