# Supplementary material for: Adaptive potential of maritime pine under contrasting environments
Source: BMC Plant Biol. 2024 Jan 9;24:37. doi: 10.1186/s12870-023-04687-w (PMC10775667; doi:10.1186/s12870-023-04687-w)
Supplement: Supplementary file 6 — Additional file 6. [file 12870_2023_4687_MOESM6_ESM.pdf]

Table S2. Characteristics of the populations of *Pinus pinaster* Ait. and the two common gardens.

Location	Code	Altitude	Longitude	Latitude	Climatic data[1]				
					**MAT**	**MTWM**	**MTCM**	**AP**	**SP**
Mimizan-FR	FA1	37	44.13417N	-1.30317W	13.28	24.80	3.20	1235	232
Pleucadec-FR	FA2	80	47.78119N	-2.34367W	11.24	21.90	2.50	804	154
Cadavedo-SP	IA1	210	43.53996N	-6.41785W	13.22	22.00	5.01	1316	204
Puerto de Vega-SP	IA2	121	43.54795N	-6.63137W	13.39	22.61	4.91	1283	194
Leiria-PT	IA3	20	39.78333N	-8.95750W	15.35	24.40	7.40	811	44
Arenas de San Pedro-SP	CS1	733	40.19482N	-5.11621W	14.18	33.41	1.24	1318	73
Coca-SP	CS2	800	41.25470N	-4.49782W	12.28	31.18	-0.57	454	77
Cuéllar-SP	CS3	830	41.37460N	-4.48403W	12.17	30.93	-0.65	468	72
Bayubas-SP	CS4	998	41.52297N	-2.87743W	10.63	29.59	-1.36	553	96
Oria-SP	SS1	1223	37.53116N	-2.35113W	13.14	30.70	0.44	357	29
Tamrabta-MO	MO1	1758	33.60000N	-5.01666W	10.66	30.40	-4.60	745	49
Cabada- SP	*HiProd*[2]	455	43.41947N	-6.53966W	12.92	23.58	3.69	1316	134
Ibias-SP	*LoProd*[3]	738	43.02157N	-6.88400W	12.94	28.22	2.17	1754	142

[1] MAT-mean annual temperature, MTWM-mean of maximum temperature of the warmest month, MTCM-mean of minimum temperature of the coldest month, AP-total annual precipitation and SP-summer precipitation- . Site climatic data is the average for the period from planting to field measurements. [2]Site index: 22 m at 20 years, northwest orientation, 75 cm average soil depth of, Lusitanean environmental zone (Metzger, 2018). [3]Site Index: 6 m at 20 years. South orientation, 20 cm average soil depth, Mediterranean Mountain environmental zone.
