# Supplementary material for: Adaptive potential of maritime pine under contrasting environments
Source: BMC Plant Biol. 2024 Jan 9;24:37. doi: 10.1186/s12870-023-04687-w (PMC10775667; doi:10.1186/s12870-023-04687-w)
Supplement: Supplementary file 7 — Additional file 7. [file 12870_2023_4687_MOESM7_ESM.pdf]

Table S3. *Number of families and trees of Pinus pinaster Ait. sampled in each of the two sites, by population.*

**Pop. Code**	***HiProd site***		***LoProd site***		**Common in the two sites**
	# Families	# Trees	# Families	# Trees	# Families
**FA1**	9	73	3	18	2
**FA2**	9	72	0	0	0
	**18**	**145**	**3**	**18**	**2**
**IA1**	10	76	10	82	10
**IA2**	5	37	3	24	3
**IA3**	10	80	5	29	3
	**25**	**193**	**18**	**135**	**16**
**CS1**	12	92	5	34	4
**CS2**	12	101	0	0	0
**CS3**	7	55	2	15	1
**CS4**	14	113	7	57	7
	**45**	**361**	**14**	**106**	**13**
**SS1**	**15**	**122**	**5**	**40**	**5**
**MO1**	**16**	**126**	**9**	**76**	**9**
**Total**	**119**	**947**	**49**	**375**	**44**
